# Functioning of a tripartite lignocellulolytic microbial consortium cultivated under two shaking conditions: a metatranscriptomic study

**DOI:** 10.1186/s13068-023-02289-0

**Published:** 2023-03-29

**Authors:** Yanfang Wang, Diego Javier Jiménez, Zhenhua Zhang, Jan Dirk van Elsas

**Affiliations:** 1 Cluster of Microbial Ecology, Groningen Institute for Evolutionary Life Sciences, University of Groningen, Groningen, The Netherlands; 2 Microbiomes and Bioenergy Research Group, Department of Biological Sciences, Universidad de los Andes, Bogotá, Colombia; 3 Department of Genetics, University Medical Center Groningen, Groningen, The Netherlands

**Keywords:** Lignocellulose, Degradation, Metatranscriptome, Bacterial–fungal consortium, Microbial interactions, CAZy, Eco-enzymology

## Abstract

**Background:**

In a previous study, shaking speed was found to be an important factor affecting the population dynamics and lignocellulose-degrading activities of a synthetic lignocellulolytic microbial consortium composed of the bacteria *Sphingobacterium paramultivorum* w15, *Citrobacter freundii* so4, and the fungus *Coniochaeta* sp. 2T2.1. Here, the gene expression profiles of each strain in this consortium were examined after growth at two shaking speeds (180 and 60 rpm) at three time points (1, 5 and 13 days).

**Results:**

The results indicated that, at 60 rpm, *C. freundii* so4 switched, to a large extent, from aerobic to flexible (aerobic/microaerophilic/anaerobic) metabolism, resulting in continued slow growth till late stage. In addition, *Coniochaeta* sp. 2T2.1 tended to occur to a larger extent in the hyphal form, with genes encoding adhesion proteins being highly expressed. Much like at 180 rpm, at 60 rpm, *S. paramultivorum* w15 and *Coniochaeta* sp. 2T2.1 were key players in hemicellulose degradation processes, as evidenced from the respective CAZy-specific transcripts. *Coniochaeta* sp. 2T2.1 exhibited expression of genes encoding arabinoxylan-degrading enzymes (i.e., of CAZy groups GH10, GH11, CE1, CE5 and GH43), whereas, at 180 rpm, some of these genes were suppressed at early stages of growth. Moreover, *C. freundii* so4 stably expressed genes that were predicted to encode proteins with (1) β-xylosidase/β-glucosidase and (2) peptidoglycan/chitinase activities, (3) stress response- and detoxification-related proteins. Finally, *S. paramultivorum* w15 showed involvement in vitamin B2 generation in the early stages across the two shaking speeds, while this role was taken over by *C. freundii* so4 at late stage at 60 rpm.

**Conclusions:**

We provide evidence that *S. paramultivorum* w15 is involved in the degradation of mainly hemicellulose and in vitamin B2 production, and *C. freundii* so4 in the degradation of oligosaccharides or sugar dimers, next to detoxification processes. *Coniochaeta* sp. 2T2.1 was held to be strongly involved in cellulose and xylan (at early stages), next to lignin modification processes (at later stages). The synergism and alternative functional roles presented in this study enhance the eco-enzymological understanding of the degradation of lignocellulose in this tripartite microbial consortium.

**Supplementary Information:**

The online version contains supplementary material available at 10.1186/s13068-023-02289-0.

## Background

Lignocellulosic biomass (LCB) is an abundant source of carbohydrates that may feed into the production of biofuels or other chemical compounds. Nearly 75% of most sources of LCB is composed of polysaccharides such as cellulose and hemicellulose [1], with lignin (aromatic polymer) constituting a third major carbonaceous compound [2]. Moreover, LCB is present in diverse organic materials and so the sources of sustainable energy feedstock are rich and diverse [3]. In light of its complexity, the deconstruction of LCB requires the action of a variety of enzymes [4], i.e., diverse lytic polysaccharide monooxygenases (LPMO’s), laccases, endoxylanases, arabinofuranosidases, glucuronidases, cellobiohydrolases, endoglucanases and β-glucosidases [3, 5]. Microbial consortia derived from natural systems with efficient LCB-degradative capacities have recently turned into research hotspots, and examples of these have been investigated by different research teams [6–9]. The use of such bottom-up approaches that aim to simplify natural biodegradative systems has resulted in effective lignocellulolytic microbial consortia [3, 10–12].

Recently, members of the bacterial genera *Sphingobacterium* and *Citrobacter* and of the fungal genus *Coniochaeta* were shown to be consistently present across lignocellulolytic consortia, with indications for their key roles in wheat straw (WS) degradation [6, 7, 13]. In detail, many *Sphingobacterium* species were found to have the potential to produce a variety of carbohydrate-active enzymes that function in LCB degradation processes, in particular endo-1,4-β-xylanases (CAZy class GH10), α-L-arabinofuranosidases (GH43) and α-L-fucosidases (GH95) [13, 14]. In addition, Citrobacter species have been reported as major scavengers of particular monomers resulting from such biodegradations [6]. Moreover, the latter organism might be capable of detoxifying the system by reducing the levels of (toxic) by-products [5], and it has been highlighted for its potential to detoxify phenolic compounds in black liquor from the pulp manufacturing industry [15]. With respect to the fungal consortium member, Mondo et al. [16] reported that *Coniochaeta* sp. 2T2.1 can efficiently degrade arabinoxylan, xyloglucan and cellulose when growing on WS. Also, promotion of lignin transformation by *Coniochaeta* sp. 2T2.1 in co-culture with the two bacteria was suggested [4]. Other strains of *Coniochaeta,* i.e., *Coniochaeta* sp. LF2, have also been reported to produce a plethora of lignocellulolytic enzymes, useful in corn stover decay processes [17].

Even though previous studies [18–20] have reported the expression of a plethora of genes encoding CAZy families in microbial consortia under different conditions, the focus was mainly on the bacterial members within the consortia. Our previous work [4] described the expression profile of CAZy families in interkingdom consortia with a focus on the fungal member. Overall, however, the functional roles within such low-complexity lignocellulolytic consortia are underexplored. A recent study performed in our lab showed that the lignocellulose-degrading activities exhibited by low-complexity microbial consortia (composed of two bacteria and one fungus) are strongly dependent on the abiotic (culture) conditions applied [21], with shaking speed acting as a major driver. In particular, fungal growth was found to be suppressed by the presence of the two bacteria at 180 rpm, whereas at 60 rpm this effect was reversed into a potential bacterial helper effect. However, the study did not examine the gene expression patterns of the members of this consortium, with respect to two questions: (1) how does shaking speed affect the interactions across the bacteria and the fungus, and (2) how do the CAZy family gene expression profiles of each strain change under different shaking conditions over time. To examine these questions, we set up an experiment in which the transcriptomes of each of the three strains in the microbial consortia were assessed, when growing on WS at two shaking speeds (60 and 180 rpm) and three time points (early-1 day, middle-5 days, late-13 days). We hypothesized that (1) shaking speed affects the interactions within the consortium by modifying the local environment, which results in behavioral changes and shifting niches; (2) the bacterial and fungal strains contribute to WS degradation with differently expressed enzymes; and (3) the enzymatic interactive synergism (different enzymes from each strain) among the three partners may change at different stages of the WS degradation process.

## Results

### Population dynamics of the consortium strains, and WS degradation performance

#### Population dynamics

**Fig. 1:**
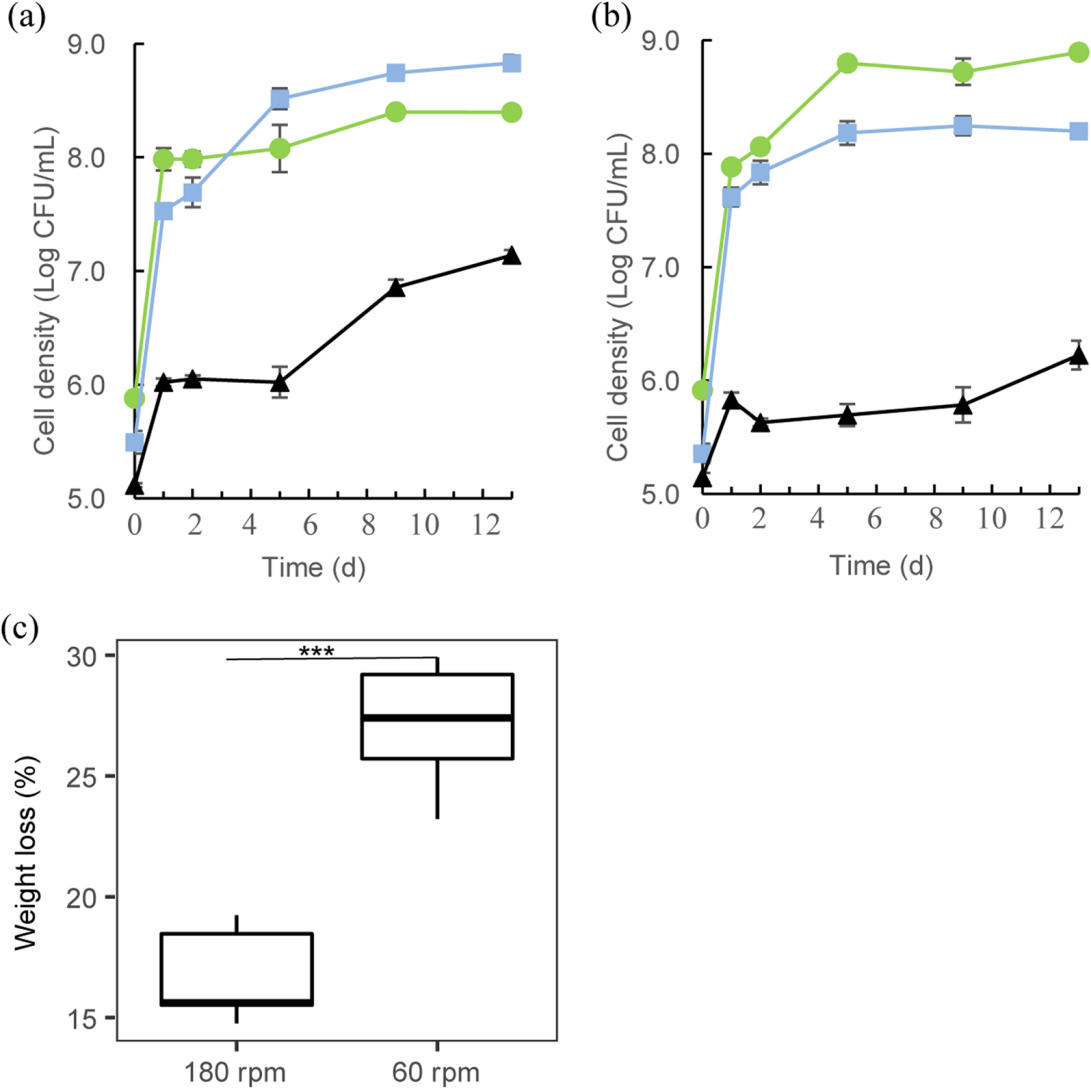
Population dynamics of *Sphingobacterium paramultivorum* w15 (blue square), *Citrobacter freundii* so4 (green circle), and *Coniochaeta* sp. 2T2.1 (black triangle) in the SWT consortium at **a** 180 rpm and **b** 60 rpm. Standard deviations are indicated by bars, or within symbol dimensions. **c** wheat straw (WS) weight loss after 13 days; asterisks (*) represent significant differences, as evidenced using Student’s T test, ***p < 0.001

In a previous study, we found differential growth effects of the three member strains of the degrader consortium, across two shaking speeds in cultures examined after 10 days [21]. Moreover, particle agglomerates were visually detected after 1 day in the cultures run at 60 rpm, but not in those at 180 rpm. The physical appearance and population dynamics data obtained in the current study, over 13 days, were consistent with these earlier data [4, 21], see Additional file 2: Fig. S1. In detail, at 180 rpm, fast initial (1 day) growth of both *S. paramultivorum* w15 and *C. freundii* so4 was followed by roughly stable or slightly increasing cell densities. The densities of both bacterial strains thus reached roughly 4–5 × 10^8^ CFU/ml. In contrast, the density of *Coniochaeta* sp. 2T2.1 propagules increased, slowly but progressively, to 1 × 10^7^ CFU/mL after 13 days (Fig. 1a). In the 60-rpm treatment, the two bacterial strains showed growth dynamics similar to that described in the foregoing, with rapid initial growth being followed by extended slow growth. Interestingly, at middle stage, *C. freundii* so4 outperformed (μ, 0.0097 ± 0.0005 h^−1^) *S. paramultivorum* w15 (μ, 0.0056 ± 0.0010 h^−1^), with cell densities (9 × 10^8^ CFU/mL) stabilizing at higher levels than those of the latter strain (2 × 10^8^ CFU/mL) (Fig. 1b). The growth of *Coniochaeta* sp. 2T2.1 could be characterized by a fast onset, followed by slowly but progressively increasing propagule numbers over experimental time. Compared to the growth at 180 rpm, *Coniochaeta* sp. 2T2.1 reached lower cell densities at 60 rpm.

#### WS degradation levels

High WS weight losses were achieved across the treatments and over time in this study, consistent with those found in the previous experiment [21]. Remarkably, the weight losses at 60 rpm (27.1 ± 2.4%) were significantly (*p* < 0.001) higher than those at 180 rpm (16.2 ± 1.7%) after 13 days (Fig. 1c).

### Gene expression profiles

To analyze the gene expression profiles of the WS-grown microbial consortia across shaking treatments and time, total mRNA was extracted from triplicate cultures at each time point and shaking speed, and cDNA was produced. Thereafter, all cDNA was sequenced. On average, 16 (± 2.6) million [bacteria] and 27 (± 7.6) million [fungus] filtered raw reads were obtained per sample (Additional file 1: Table S1). Principal components analysis (PCA) showed that the expression profiles were consistent across the replicates, as all replicates per treatment clustered together (Additional file 2: Fig. S2). The gene expression profiles of each strain in the consortium, at the predefined levels “low” (< 100 Transcripts Per Kilobase Million (TPM)), “intermediate” (100–1000 TPM) and “high” (> 1000 TPM), are summarized in Table 1. For the two bacteria, a majority (54–73%) of genes was expressed at intermediate level. In contrast, the great majority (92.5%) of the fungal genes was expressed at low level. For all three members of the consortium, most genes expressed at high level were growth- or substrate attack-related. In the further analyses, we placed a focus on the expression of (1) growth- and adhesion-associated genes, (2) genes enabling carbohydrate attack (using the CAZy database as a reference), and (3) genes with otherwise potentially supportive roles.

**Table 1:** Overall gene expression at different levels across strains under different shaking speeds over time

	TPM value	Level	180 rpm			60 rpm			Ratio (%)
			1d	5d	13d	1d	5d	13d	
so4 (4567)	> 1000	H	61	53	35	67	25	45	1.04
	100–1000	I	1151	1873	1333	2476	3976	4216	54.83
	< 100	L	3356	2641	3199	2024	566	306	44.13
w15 (5497)	> 1000	H	62	41	49	56	40	28	0.84
	100–1000	I	3731	3556	4478	3364	4079	4956	73.26
	< 100	L	1704	1900	970	2077	1378	513	25.90
2T2.1 (23,406)	> 1000	H	128	69	62	118	63	59	0.36
	100–1000	I	1491	1742	1770	1588	1632	1780	7.12
	< 100	L	21,787	21,595	21,574	21,700	21,711	21,567	92.52

Transcripts Per Kilobase Million (TPM) values of each sample were used. Expression level: high (H), intermediate (I), and low (L). Ratio calculated as: mean of expressed gene number at a certain level/ total expressed gene number of each genome (number in brackets under the strain name). Genes with raw read numbers in each sample below 10 or with summed number in all 18 samples below 60 were treated as non-expressed

### Expression of growth- and adhesion-associated genes

The details of expression of selected genes with potential relevance for WS-driven growth of each strain over time at two shaking speeds can be found in Additional file 2. First, the high expression levels (1,984 ± 27–874 ± 104 TPM) of the exponential growth indicator *rpoD* gene of *S. paramultivorum* w15 at early and middle stages, and the decrease at late stage, indicated that strain w15 grew rapidly early on and reached stationary phase as from middle growth stage, at both shaking speeds. Moreover, the significant increase (Log2-fold change = 2.0 ± 0.2, *p*_*adj*_ < 0.001), from 2,418 ± 494 to 12,759 ± 815 TPM, of the *dps* (stationary phase-induced gene) transcript numbers at late stage at 180 rpm indicated that strain w15, at this point in time, responded strongly to conditions of (starvation) stress (Additional file 2: Fig. S3). As for *C. freundii* so4, the high expression level of stationary phase-induced gene *elaB* at early stage (2,473 ± 385 TPM at 180 rpm, and 2,072 ± 28 TPM at 60 rpm), next to its lowering at later stages (Log2-fold change = 2.9 ± 0.2, *p*_*adj*_ < 0.001), indicated that a major part of the population was in need of oxidative stress protection (potentially a spin-off of rapid metabolism); however, this effect became somewhat less dominant in later stages. Remarkably, the strain so4-specific aerobic respiration regulatory gene *arcA* and the anaerobic regulatory *fnr* gene showed different trends across the two shaking speeds: at late stage, the expression of the *arcA* gene (encoding aerobic respiration control protein ArcA) was significantly lower (Log2-fold change = 1.4 ± 0.2, *p*_*adj*_ < 0.001) at 60 (474 ± 27 TPM) than at 180 rpm (730 ± 20 TPM). In contrast, the *fnr* gene (encoding anaerobic regulatory protein FNR) was higher expressed (Log2-fold change = 0.4 ± 0.1, *p*_*adj*_ < 0.001) at 60 than at 180 rpm (427 ± 10 versus 184 ± 25 TPM). Thus a switch to flexible (aerobic/microaerophilic/anaerobic) metabolism was postulated to take place at 60 rpm, probably in a major part of the *C. freundii* so4 population (Additional file 2: Fig. S3). Finally, examination of the phylogenetic placement of *Coniochaeta* sp. 2T2.1 genes encoding “cell adhesion complex” protein bystin (KOG3871) and adhesion glycoprotein fasciclin (KOG1437) provided an indication for the potential involvement of these proteins in cell clumping and/or adhesion processes (see Discussion section). In detail, such (glyco) proteins may make part of the *Coniochaeta* sp. 2T2.1 cell wall, promoting adherence to surfaces. Two out of eight strain 2T2.1 adhesion glycoprotein genes (GE09DRAFT_1064529 and GE09DRAFT_1222170), showed similar expression levels at early and middle stages across the two shaking speeds, but were significantly higher expressed (Log2-fold change > 1.1 ± 0.2, *p*_*adj*_ < 0.001) at 60 (390 ± 15 TPM) than at 180 rpm (175 ± 34 TPM) at late stage. Interestingly, another adhesion glycoprotein gene (GE09DRAFT_1202287) was significantly lower expressed (Log2-fold change = 1.2 ± 0.2, *p*_*adj*_ < 0.001) at early and middle stages at 60 vs 180 rpm, but higher expressed (Log2-fold change = 1.3 ± 0.1, *p*_*adj*_ < 0.001) at late stage. These data indicated that *Coniochaeta* sp. 2T2.1 expresses genes that are potentially involved in cell aggregation and adhesion differentially, but preferentially at 60 rpm, as compared to 180 rpm.

### Expression of genes encoding CAZy family proteins

Data from previous studies performed in our laboratory [3–5, 16] clearly pointed to particular CAZy-encoding genes of the bacterial (*S. paramultivorum* w15 and *C. freundii* so4) and fungal (*Coniochaeta* sp. 2T2.1) consortium members, with predicted key roles in WS degradation processes. Hence, these genes, listed in Table 2, next to others that stood out given their expression patterns, were bookmarked for further examination. In the following section, we briefly describe the expression patterns of these genes and highlight those that were highly or differentially expressed over time. We start with the trends found under ‘standard’ condition (180 rpm), then describe the trends at 60 rpm, and finally do a comparative analysis of the expression patterns between the two shaking speeds.

**Table 2:** Genes predicted to encode proteins of different CAZy classes and their numbers within the genome of members of the SWT consortium

CAZy	Activity	Substrate	2T2.1	w15	so4	References
AA16	LCMO^a^	Cellulose	6	0	0	
GH16	Glucanase	Cellulose	40	10	0	[5, 16], H-T^b^
GH32	β-Fructofuranosidase	Fructan	9	3	2	
GH128	β-1,3-Glucanase	Cellulose	8	0	0	[16], H-T
GH5	Endo-glucanase	Cellulose	36	1	2	[3]
GH71	α-1,3-Glucanase	Cellulose	13	0	0	[4]
GH7	Cellobiohydrolase	Cellulose	12	0	0	[4, 16], H-T
AA9	LCMO	Cellulose	38	0	0	H-T
GH10	Xylanase	Hemi^c^	14	2	0	H-T
GH11	Endo-β-xylanase	Hemi	13	0	0	[4, 16]
GH30	Endoxylanase	Hemi	10	3	0	[3, 16]
CE1	Acetyl xylan esterase	Hemi	21	11	2	[4, 16]
CE5	Acetyl xylan esterase	Hemi	14	0	0	H-T
GH43	β-Xylosidase/α-L-arabinofuranosidase	Hemi	40	17	1	[4, 5, 16], H-T
GH93	Exo-α-L-1,5-arabinanase	Hemi	6	0	0	[4, 16]
GH3	β-Xylosidase/β-glucosidase	NA	28	6	4	[4, 5]
GH1	β-Glucosidase/galactosidase	NA	4	1	4	[5, 16]
GH51	α-L-Arabinofuranosidase	Hemi	4	3	0	[4, 16]
GH62	α-L-Arabinofuranosidase	Hemi	3	0	0	[4, 16]
GH29	α-L-Fucosidase	NA	0	16	0	[5]
GH95	Fucosidase/galactosidase	NA	2	10	0	[5]
GH2	β-Galactosidase	NA	12	20	2	[5]
GH27	α-Galactosidase	NA	6	0	0	H-T
GH92	α-Mannosidase	NA	8	10	0	[3, 5]
GH74	Xyloglucanase	Hemi	2	0	0	
AA3		Lignin	9	4	0	
AA3_2	AAO^d^/glucose oxidase	Lignin/cellulose	19	0	0	[4, 16]
CE15	Lignin esterase	Lignin	7	0	0	
AA1_3	Laccase	Lignin	5	0	0	[4]
AA1_2	Laccase	Lignin	5	0	0	[4]
AA3_3	Alcohol oxidase	Lignin	5	0	0	[16]
GH23	Peptidoglycan lyase	NA	0	2	6	
GH73	Peptidoglycan hydrolase	NA	0	2	2	
GH79	β-Glucuronidase	Proteoglycan	7	0	0	[4]
GH67	α-Glucuronidase	Proteoglycan	2	1	0	
GH88	β-Glucuronyl hydrolase	Proteoglycan	7	5	2	
GH13	α-Amylase	Starch	25	6	6	

^a^: LCMO: lytic cellulose monooxygenase; ^b^: H-T: high TPM in *Coniochaeta* sp. 2T2.1; ^c^: Hemi: hemicellulose; ^d^: AAO: aryl alcohol oxidase

### Expression at high shaking speed (180 rpm)

In this section, we describe the results of the gene expression measurements within the consortia, focusing on the dynamics per strain. As outlined in the foregoing, we start by describing the expression patterns at 180 rpm, after which we address those at 60 rpm, and the differences between the two shaking speeds.

#### S. paramultivorum w15

**Fig. 2:**
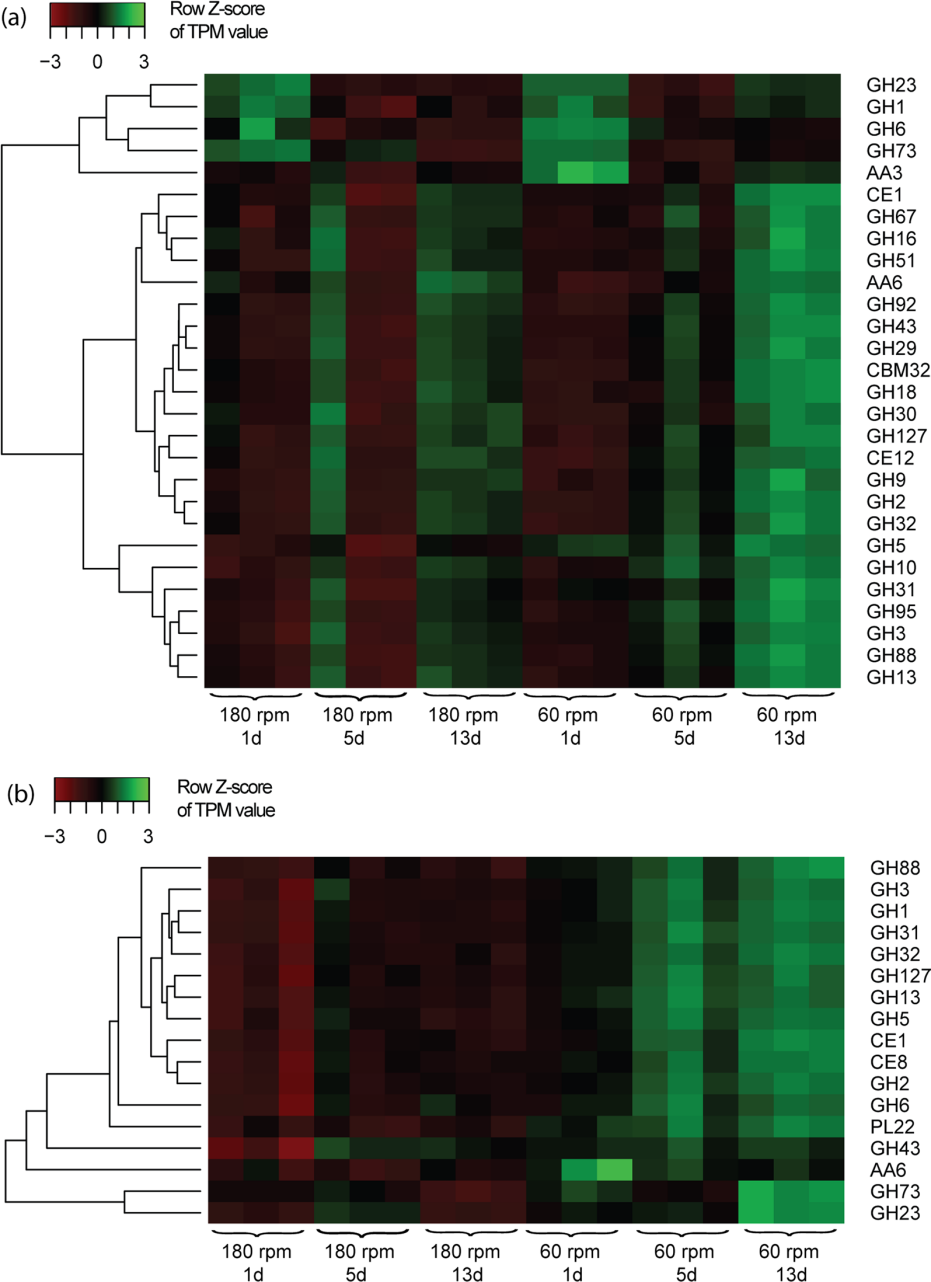
Heat map using row Z-score of TPM (Transcripts Per Kilobase Million) values of CAZy-related transcripts (rows) from **a** *Sphingobacterium paramultivorum* w15 and **b** *Citrobacter freundii* so4 growing in a tripartite microbial consortium (+ *Coniochaeta* sp. 2T2.1) at 180 rpm and 60 rpm over 13 days using wheat straw as the sole of carbon and energy source

The analyses showed broad involvement of *S. paramultivorum* w15 in hemicellulose hydrolysis processes versus more limited involvement in cellulolytic activities. Clearly, *S. paramultivorum* w15 expressed genes encoding seven hemicellulose-degrading enzymes quite stably over experimental time. These were genes predicted to attack the WS xylan backbone (endoxylanases of CAZy class GH30), and to cut off xylan side chains (α-L-arabinofuranosidases of CAZy classes CE1, GH43 and GH51). In addition, *S. paramultivorum* w15 stably expressed genes encoding enzymes that, presumably, hydrolyze galactoglucomannan (GH2, GH92) and xyloglucan (GH95, GH29), at consistently high levels (TPM > 1000) over time (Fig. 2a). Also, an increase of expressed genes encoding enzymes classified as β-xylosidase/β-glucosidase (GH3), from 695 ± 52 to 876 ± 29 (*p* < 0.01) TPM, was observed over time. This finding may indicate progressively increasing investment in the debranching of hemicellulose. Overall, the expression of genes encoding proteins of CAZy families GH2, GH92, GH43, CE1, GH29 and GH95 in *S. paramultivorum* w15 stayed at high level from early to middle stages, even increasing afterwards (Fig. 2a), indicating that strain w15 has a key and stable role in degrading specific parts of the WS hemicellulose moiety. On the other hand, only few genes encoding cellulolytic enzymes (CAZy classes GH5, GH9 and GH16) were expressed by *S. paramultivorum* w15 (Table 2). In detail, a gene encoding a GH16 family protein was initially expressed at high level (1200 ± 110 TPM) at early and middle stages, being even higher (1,348 ± 40 TPM, *p* > 0.05) at late stage. In contrast, the expression of the gene for a GH5 protein was initially low (~ 92 ± 17 TPM) and remained at this level over experimental time (Additional file 3: Table S3). A putative lignin transformation-related gene (cellobiose dehydrogenase/aryl alcohol oxidase, class AA3) was expressed at around 574 ± 27 TPM at early stage, and this level was roughly maintained over experimental time.

#### C. freundii so4

Limited involvement in lignocellulytic activities was found in *C. freundii* so4, as a result of its genomic features, as partly shown in Table 2. In detail, one gene encoding a GH5-type protein (with presumed cellulase activity) was stably expressed at around 145 ± 26 TPM, from early to late stages. Furthermore, two genes encoding enzymes of CAZy families GH2 and GH43 (involved in hemicellulose degradation) were expressed at around 142 ± 21 TPM, over experimental time. Remarkably, strain so4 expressed several genes associated with oligosaccharide catabolism (CAZy classes GH1, GH3) at rather stable intermediate levels (range 300–600 TPM) (Fig. 2b). Expressed genes encoding enzymes potentially involved in lignin transformation were not detected in *C. freundii* so4, due to the absence of these genes from its genome as shown in Table 2. A gene encoding a protein presumably involved in peptidoglycan consumption (peptidoglycan lyase/ chitinase; GH23 with CBM50) was also active, with its expression increasing from early (589 ± 39 TPM) to middle stages (1,021 ± 54 TPM), decreasing afterwards (549 ± 21 TPM). Similar expression dynamics was found for a gene encoding a peptidoglycan hydrolase (known as lysozyme/ peptidoglycan lytic transglycosylase/chitinase; GH73). This gene was expressed at middle level (251 ± 1–273 ± 29 TPM) at early and middle stages, decreasing to 186 ± 8 TPM at late stage (Additional file 2: Fig. S4).

#### Coniochaeta sp. 2T2.1

**Fig. 3:**
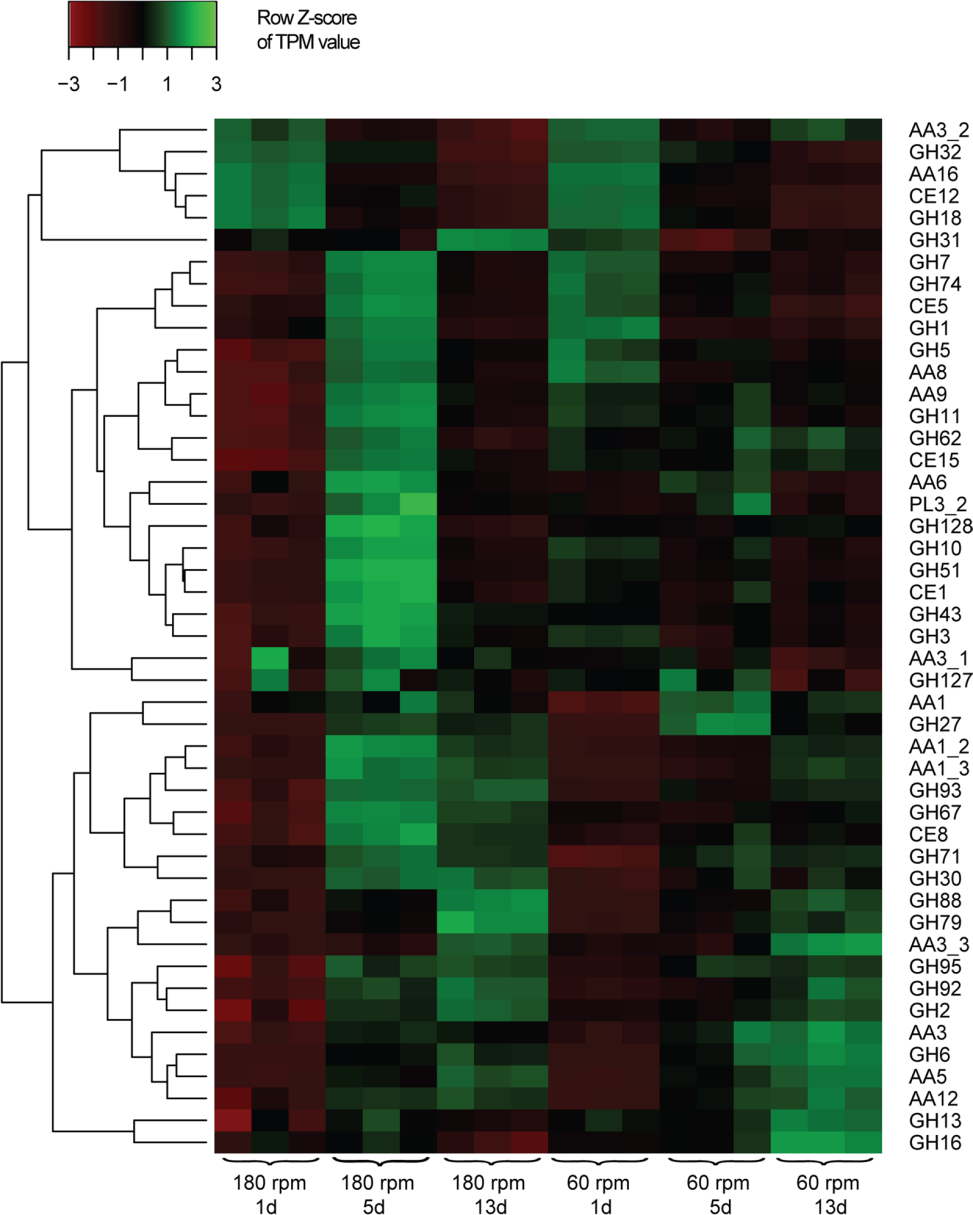
Heat map using row Z-score of TPM (Transcripts Per Kilobase Million) values of CAZy-related transcripts (rows) from *Coniochaeta* sp. 2T2.1 growing in a tripartite microbial consortium (+ *Sphingobacterium paramultivorum* w15 and *Citrobacter freundii* so4) at 180 rpm and 60 rpm over 13 days using wheat straw as the sole of carbon and energy source

The transcriptome data showed that *Coniochaeta* sp. 2T2.1 quickly became involved in the degradation of both the WS cellulose and hemicellulose moieties. In early stage, two genes encoding cellulolytic enzymes were found to be highly active (TPM > 1000). These were (1) a gene associated with lytic cellulose monooxygenase (LCMO; class AA16; concomitant with the absence of AA16 from the two bacteria) (Table 2), and (2) a gene predicted to encode an endoglucanase (GH16; involved in the breakdown of low-crystallinity cellulose, creating free chain ends) (Fig. 3). The expression of the former gene decreased gradually over time, while that of the latter one stayed at high level till middle stage (1347 ± 76 TPM), decreasing at late stage (942 ± 123 TPM). Moreover, these two genes ranked high among the top-200 genes (TPM > 703.2 ± 84.6) in early stage. In addition, genes encoding five other cellulolytic CAZy family proteins were expressed at intermediate levels at early stage; these were genes encoding endoglucanases of CAZy classes GH128, GH5 and GH71, a gene for a cellobiohydrolase (GH7) and one for an LCMO (AA9) (Fig. 3). The expression dynamics of these five genes showed similar trends, with high levels at the middle stages (Fig. 3). The strong investment in expression of AA9 and GH7 type enzymes at middle stage at 180 rpm confirmed the importance of those enzyme families in the degradation of the cellulose moiety of WS by *Coniochaeta* sp. 2T2.1 (Fig. 3).

Regarding the expressed genes involved in hemicellulose degradation, mainly transcripts of genes encoding proteins with activities on (the main xylan moiety) arabinofuranoxylan were detected. At early stage, a high percentage (42%; 8/19) of these genes was expressed at intermediate level. Specifically, the expressed genes were found to encode endo-β-xylanases (GH11, GH10—hydrolyze xylosidic linkages in xylans), acetyl xylan esterases (CE1, CE5) that catalyze the hydrolysis of acetyl groups from xylan, and α-L-arabinofuranosidase (GH43), arabinanase (GH93), xylosidase and glucosidase (GH3, GH1) that work on side chains of xylan, as well as downstream. The expression of all of these genes showed similar dynamics, with highest expression levels at middle stages, decreasing afterwards (Fig. 3). In contrast, genes encoding GH11 (endohydrolyzing xylosidic linkages), CE5 (hydrolyzing acetyl groups from xylan), and GH93 (attacking arabinose side chains) type proteins stayed at high expression levels till late stage (Fig. 3). Finally, genes encoding enzymes potentially hydrolyzing pectin (CE8, PL3_2), mannan (GH92) and xyloglucan (GH74) were lowly expressed over time (Additional file 3: Table S3).

Of seven *Coniochaeta* sp. 2T2.1 genes encoding lignin-transforming enzymes, five were initially expressed at low levels. These were genes for laccase (AA1_2; AA1_3), lignin esterase (CE15), copper radical oxidase (AA5) and a putative aryl alcohol oxidase/cellobiose dehydrogenase (AA3) (Fig. 3). The remaining two, encoding an aryl alcohol oxidase (AA3_2) and an alcohol oxidase (AA3_3) were at intermediate levels at early stage (Fig. 3). At middle stage, the expression of the genes encoding laccases (AA1_3; AA1_2) increased significantly, for AA1 3 to high level (from 69 ± 2 to 1305 ± 369 TPM). This coincided with the increasing expression of the gene encoding the lignin esterase (CE15); see Fig. 3. Moreover, the expression of the alcohol oxidase (AA3_3) gene increased from 182 ± 25 (early), via 220 ± 44 (middle), to 809 ± 59 TPM (late stage, Fig. 3).

### Expression at low shaking speed (60 rpm)

#### S. paramultivorum w15

The trends in the expression patterns of the (hemi)cellulolytic genes of *S. paramultivorum* w15 at 60 rpm were similar to those at 180 rpm (see above), however the expression levels at 60 rpm were consistently higher (*p* > 0.05 at early and middle stages; *p* < 0.05 at late stage). Remarkably, the expression level of the putative lignin transformation associated gene (AA3) of *S. paramultivorum* w15 at 60 rpm was relatively high throughout (*p* < 0.05 at early and late stages; *p* > 0.05 at middle stages), ranging from 552 ± 48 (middle stage) to 878 ± 82 TPM (early; Fig. 2a).

#### C. freundii so4

At 60 rpm, the expression dynamics of the genes encoding GH1, GH3, GH2, GH43 and GH13 class enzymes in *C. freundii* so4 was similar to that at 180 rpm, however the expression levels were generally raised (*p* < 0.05, except for GH43 at middle and late stages). This indicated similar involvement in the WS degradation and ensuing processes of *C. freundii* so4 between 60 and 180 rpm. The expression of the genes encoding GH23-like proteins gradually increased over time, from early (871 ± 89 TPM) via middle (915 ± 94 TPM) to late stage (1700 ± 161 TPM). Finally, genes encoding GH73-like proteins were expressed at 316 ± 29 TPM (early stage), 251 ± 15 TPM (middle stage) and 446 ± 27 TPM, at late stage (Additional file 2: Fig. S4).

#### Coniochaeta sp. 2T2.1

At early stage at 60 rpm, the two genes encoding CAZy class GH16 and AA16 proteins showed high expression levels (> 1000 TPM). In addition, genes encoding proteins of CAZy families CE5, CE1, GH11, GH10 (all associated with xylan decomposition) and GH7 and AA9 (attacking cellulose) were also expressed at high levels at early stage (Fig. 3). The expression of all these genes (encoding proteins of six CAZy families) decreased afterwards, however some (encoding proteins of classes CE5, GH11 and AA9) stayed at high level till late stage (Fig. 3). The remainder (encoding CAZy class GH7, CE1 and CE10 proteins) stayed at intermediate levels. Lignin transformation-related genes (AA1, AA5, AA3_1) were expressed similarly at low levels (4–30 TPM) over time. The gene for a lignin esterase (CE15) was expressed at intermediate level (171 ± 34 TPM) at early stage (Fig. 3), maintaining this level till late stage (Fig. 3). Genes encoding CAZy family AA3 and AA1_2 proteins were expressed at low level at early stage (50 ± 6 TPM), then increased to intermediate level at middle (161 ± 90 TPM) and late stages (270 ± 55 TPM). A similar trend was observed for the gene encoding AA1_3 proteins, which was expressed at low level at early stage (66 ± 2 TPM), increasing to 109 ± 24 TPM at middle stage and 471 ± 81 TPM at late stage. The expression of AA3_3 genes increased from 274 ± 30 TPM at early stage, via 286 ± 85 TPM at middle stage, to 1,308 ± 203 TPM at late stage.

### Comparison between high and low shaking speed

To uncover differences in CAZy gene expression patterns across the two shaking speeds, we performed paired comparisons at each time point between 180 and 60 rpm using DESeq2 (*p*_*adj*_-value  < 0.05, Wald test; Log2-fold change  ≥ 1, see Methods). All of the aforementioned genes (Table 2) predicted to encode CAZy family proteins in *S. paramultivorum* w15, *C. freundii* so4 and *Coniochaeta* sp. 2T2.1 were taken as the targets.

#### S. paramultivorum w15

Overall, the majority of the strain w15 genes encoding CAZy family proteins (Table 2) was expressed at similar levels (Log2-fold change  < 1) at similar sampling times across the two shaking speeds. This, combined with the similar population dynamics (Fig. 1), indicated that the role of *S. paramultivorum* w15 attacking WS hemicellulose was relatively independent of shaking speed. However, there were some exceptions. One gene (gene ID: GJ692_RS22460, encoding a peptidoglycan hydrolase; GH73) showed differential expression levels across the shaking speeds. In middle stage, its expression was higher (Log2-fold change = 1.5 ± 0.3, *p*_*adj*_ < 0.001) at 180 than at 60 rpm. Furthermore, albeit with slightly lower Log2-fold change (Log2-fold change ~ 0.91, 0.88), at early stage, the expression of two genes encoding CAZy family AA3 proteins (potentially involved in lignin transformations) was significantly (*p*_*adj*_ < 0.001) higher at 60 than at 180 rpm, suggesting a possibly enhanced role of *S. paramultivorum* w15 in lignin transformation at lower shaking speed (Fig. 2).

#### C. freundii so4

Interestingly, some genes predicted to encode cell wall attacking enzymes that showed raised expression levels at the two shaking speeds were different. The gene encoding a GH23 class enzyme (gene ID: GJ690_RS02100) [being expressed similarly, at 202 ± 23–318 ± 52 TPM, at early stage at both shaking speeds] was significantly higher expressed (Log2-fold change = 1.5 ± 0.2, *p*_*adj*_ < 0.001) at middle stage at 180 rpm (514 ± 24 TPM) than at 60 rpm (240 ± 42 TPM). However, this reversed at late stage (Log2-fold change = 2.3 ± 0.2, *p*_*adj*_ < 0.001), giving 910 ± 182 TPM (60 rpm) versus 103 ± 3 TPM (180 rpm). Another gene, encoding a chitin-binding CBM5 (gene ID: GJ690_RS07125) was expressed at 159 ± 17–254 ± 54 TPM at early and middle stages at both shaking speeds, yet it was significantly higher expressed (Log2-fold change = 1.1 ± 0.1, *p*_*adj*_ < 0.001) at 60 rpm (676 ± 47 TPM) than at 180 rpm (181 ± 8 TPM) at late stage. These data indicated that there could be a competition for substrate between *C. freundii* so4 and *Coniochaeta sp.* 2T2.1, becoming more intense at late stage at 60 rpm.

#### Coniochaeta sp. 2T2.1

**Fig. 4:**
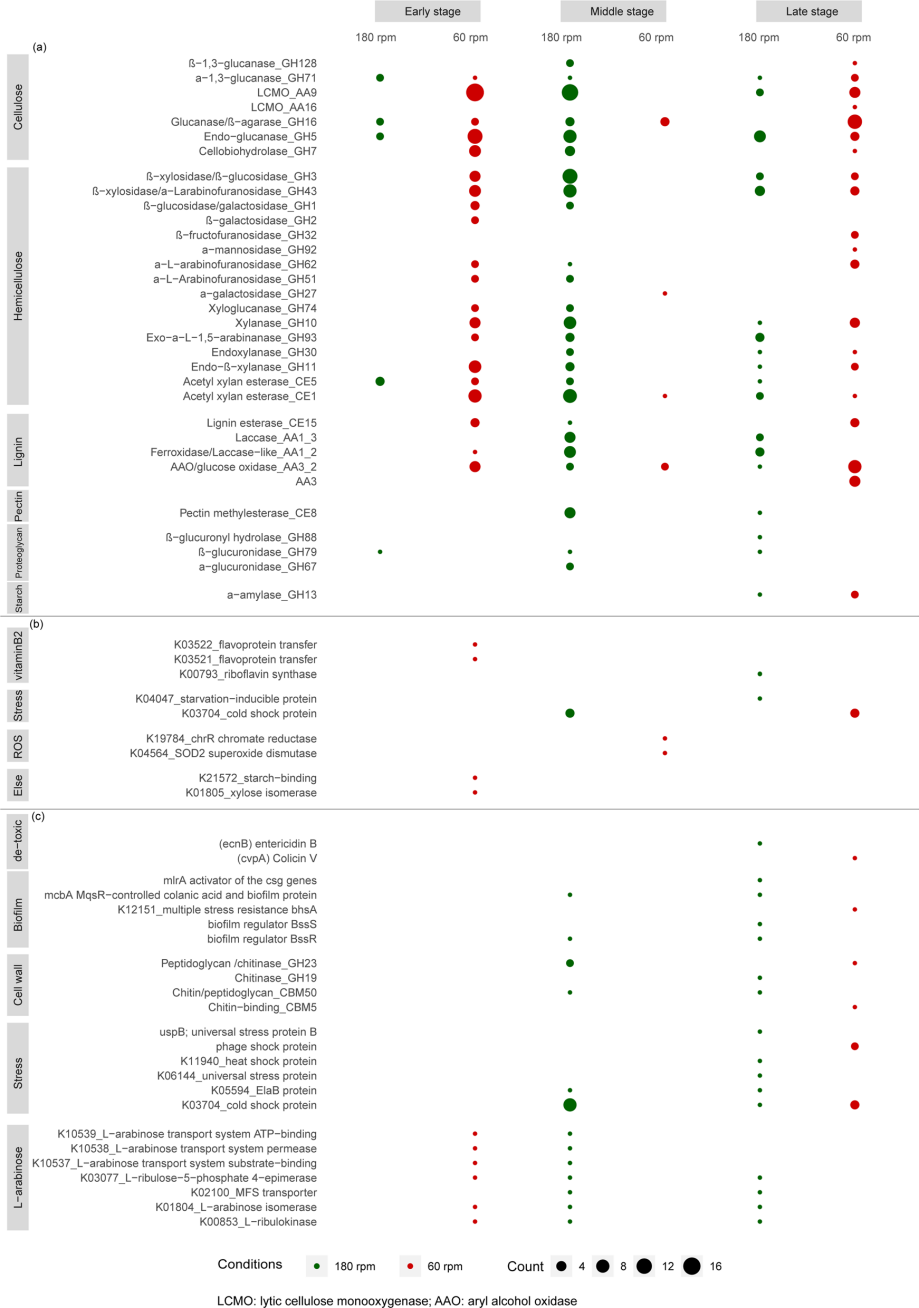
Number of genes significantly upregulated between 180 rpm (green) and 60 rpm (red) of **a** *Coniochaeta* sp. 2T2.1, **b** *S. paramultivorum* w15 and **c** *C. freundii* so4 at each time point. The size of the bubble is related to the number of significantly upregulated genes

Large gene sets encoding (hemi) cellulose- and lignin-attacking proteins were more highly expressed at 60 than at 180 rpm. For example, at early stage, 97 such genes fell in this class, especially those encoding enzymes attacking cellulose (AA9, GH7), arabinoxylan (GH10, GH11, CE1, GH43, GH51, GH62) and lignin (AA1_2, CE15, AA3_2) (Fig. 4). At middle stage, a larger number of (hemi) cellulose- and lignin-attacking genes was higher expressed at 180 rpm (113) than at 60 rpm (10). The ligninolytic activity was also remarkable, given the expression of a gene for alcohol oxidase (AA3_3) at ~ 200 TPM from early to middle stages at both shaking speeds, which increased to 1,308 ± 203 TPM at 60 rpm, as compared to 809 ± 59 TPM at 180 rpm (Additional file 3: Table S3). Interestingly, genes encoding enzymes that may hydrolyze arabinogalactan-proteins/proteoglycans (GH79, GH67, GH88) were significantly higher (*p* < 0.01) expressed at late stage at 180 rpm than at 60 rpm (Additional file 2: Fig. S5). The relatively low expression of these genes at 60 rpm might leave more proteoglycans attaching to the cell wall, which is consistent with the adhesion observed at 60 rpm.

### Other functions that potentially affect consortium behavior

We further examined several selected differentially expressed genes of *S. paramultivorum* w15 and *C. freundii* so4 between two shaking speeds, where appropriate, compared to the corresponding *Coniochaeta* sp. 2T2.1 genes. This might lead us to potential key functions of the two bacteria in the consortium at the different shaking speeds. Hereunder, these genes are examined and discussed.

#### Vitamin B2 generation and metabolism in the SWT consortium

**Fig. 5:**
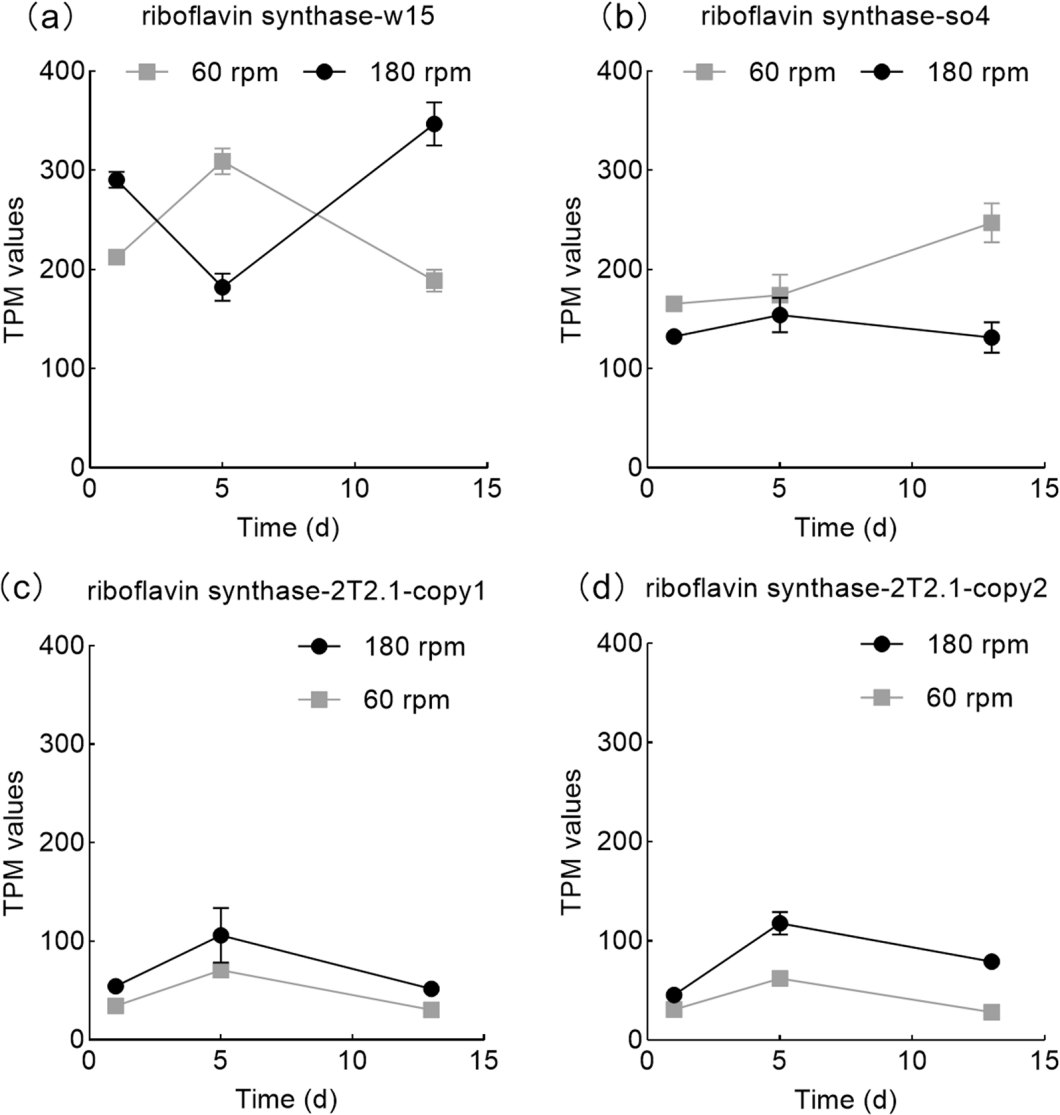
Expression dynamics of genes encoding vitamin B2 (riboflavin) synthase in **a** *S. paramultivorum* w15, **b** *C. freundii* so4, **c** and **d** *Coniochaeta* sp. 2T2.1 (2 copies). Standard deviation of the triplicates indicated as error bars

At early stage, two genes, denoted *fixA* and *fixB* (predicted to encode the alpha and beta subunits of a flavoprotein involved in electron transfer -contains a nucleic acid derivative of riboflavin/vitamin B2), were significantly higher expressed (Log2-fold change = 1.1 ± 0.1, *p*_*adj*_ < 0.001) at 60 than at 180 rpm (Fig. 4b). In addition, a single gene, *ribE* (part of the top-200 expressed genes, at middle stage at 60 rpm), encoding a riboflavin synthase (K00793) in *S. paramultivorum* w15 showed differential expression dynamics across two shaking speeds. At 180 rpm, its expression decreased from early (291 ± 8 TPM) to middle (182 ± 14 TPM) stages, then increasing to 347 ± 22 TPM at late stage. At 60 rpm, it increased from early (212 ± 5 TPM) to middle (309 ± 13 TPM) stages, then decreasing to 189 ± 11 TPM (Fig. 5a). At late stage, this gene was significantly higher expressed (Log2-fold change = 1.0 ± 0.1, *p*_*adj*_ < 0.001) at 180 than at 60 rpm.

Interestingly, gene *ribE* was also found in *C. freundii* so4 (1 copy, 37.97% similarity to *ribE* of strain w15) and *Coniochaeta* sp. 2T2.1 (2 copies; internal similarity 98%, with amino acid similarity to the paralog of *S. paramultivorum* w15 of around 35–36%). *C. freundii* so4 showed stable *ribE* expression levels between the early and middle stages (120–160 TPM), with—at late stage—the expression increasing at 60 rpm (247 ± 20 TPM), versus a decrease at 180 rpm (132 ± 15) (Fig. 5b). The two *ribE* genes in *Coniochaeta* sp. 2T2.1 showed expression dynamics at both shaking speeds similar to those of *S. paramultivorum* w15 at 60 rpm, with expression levels in the range 50–110 TPM (Fig. 5cd).

#### Reactive oxygen species (ROS) protection: role of S. paramultivorum w15 at both shaking speeds

At middle stage, two selected *S. paramultivorum* w15 genes encoding (1) a chromate reductase (*chrR*, K19784) and (2) a superoxide dismutase (*SOD2*, K04564)—both involved in oxidative stress defenses—were significantly higher expressed (Log2-fold change = 1.2 ± 0.4, *p*_*adj*_ < 0.001) at 60 than at 180 rpm (Fig. 4b). Superoxide dismutase catalyzes the conversion of superoxide into oxygen and hydrogen peroxide, controlling the ROS level. This may also contribute to lignin transformations. These genes ranked, differentially, in the top-200 expressed genes at early (both shaking speeds; *SOD2*), middle (60 rpm; both) and late stages (60 rpm; *chrR*). Hence, we surmised that *S. paramultivorum* w15 invests strongly in ROS protection processes primarily in the low-shaking-speed treatment (Additional file 2: Fig. S6).

#### Detoxification: role of C. freundii so4 at late stage different between shaking speeds

The gene *ecnB,* encoding entericidin B (a compound that is potentially involved in detoxification responses), was highly active at two shaking speeds from early (TPM > 1500) to middle stages (785 ± 60–855 ± 77 TPM), followed by a decrease to 428 ± 45 TPM (180 rpm) and 157 ± 14 TPM (60 rpm) (Additional file 2: Fig. S7a). A second gene, *cvpA,* encoding a Colicin V like protein (a small extracellular proteinaceous toxin, killing sensitive almost-kin cells by disrupting membranes), was expressed at similar levels (~ 150 TPM) from early to middle stages at both shaking speeds, but was significantly higher expressed (Log2-fold change = 1.5 ± 0.1, *p*_*adj*_ < 0.001) at late stage at 60 rpm (541 ± 51 TPM) than at 180 rpm (111 ± 13 TPM) (Additional file 2: Fig. S7b).

#### Biofilm regulation and stress alleviation: role of C. freundii so4

Several strain so4 genes involved in stress responses were found to be expressed at similarly high levels (> 1000 TPM) at early stage across shaking speeds. These genes yielded higher expression levels at middle and late stages, at 180 rpm. Some of these genes were predicted to produce proteins involved in biofilm formation processes, e.g., the biofilm regulators encoded by genes *bssR* and *bssS,* which can repress biofilm formation [22]. Also, the *uspB* gene (encoding universal stress protein B) was expressed more strongly at 180 versus 60 rpm, indicating higher stress and less biofilm formation at 180 rpm (Additional file 2: Fig. S8).

Interestingly, gene *bhsA,* encoding the BhsA protein (involved in multiple stress resistance; decreasing aggregation and cell surface hydrophobicity) was expressed at middle level (500–700 TPM) from early to late stages at both shaking speeds, being significantly higher expressed (Log2-fold change = 1.4 ± 0.3, *p*_*adj*_ < 0.001) at late stage at 60 versus 180 rpm (2,946 ± 881 versus 622 ± 24 TPM) (Additional file 2: Fig. S8). Moreover, gene *mcbA* (MqsR-controlled colanic acid and biofilm protein A; repressing biofilm formation; [23]), was expressed at very high levels at early stage (3,168 ± 859 TPM at 180 rpm, 2,419 ± 125 TPM at 60 rpm; Log2-fold change = 0.7 ± 0.2,* p*_*adj*_ < 0.001); it was significantly higher expressed at 180 compared to 60 rpm at middle (1,647 ± 380 vs 498 ± 35 TPM, Log2-fold change = 2.1 ± 0.2, *p*_*adj*_ < 0.001) and late stages (2,430 ± 205 vs 292 ± 13 TPM, Log2-fold change = 3.8 ± 0.2, *p*_*adj*_ < 0.001).

## Discussion

In this study, we examined the gene expression patterns of three strains of a WS-grown microbial consortium across two shaking speed conditions over time. We here examine the salient features of the consortia regarding growth dynamics, WS degradation and consortium-“support” related genes.

### The behavior of *S. paramultivorum* w15, *C. freundii* so4 and *Coniochaeta.*sp. 2T2.1 differed between two shaking conditions

In line with our recently published data [21], we clearly observed the behavior (population dynamics) of all three strains to be different across the two shaking speeds that were employed (Fig. 1). The genome information related to the three members of the consortium, as described in our previous work [5, 16], was used as a reference for the gene transcript measurements. Clearly, the gene expression patterns over time, which revealed both similarities and differences across shaking speeds, were backed up with predicted function data, as is further discussed in detail below.

### *S. paramultivorum* w15

*S. paramultivorum* w15, as a strictly aerobic bacterium, revealed rapid growth, up to cell numbers in the 10^8^ CFU/ml range, at 180 as well as 60 rpm (Fig. 1). This indicated fair niche occupancy by this organism, in other words, a definite role in the WS-grown consortium. The fast growth of strain w15 at both shaking speeds may relate to a potential positive “priority effect”, indicating that strain w15 is postulated to quickly access the WS and decompose it, primarily by attacking on its arabinoxylan moiety. Considering the fact that the majority (> 58%, [5]) of hemicellulose-degrading CAZy families from *S. paramultivorum* w15 are secreted proteins, such attacks by released enzymes may have resulted in the opening up of suitable niches for the companion organisms *C. freundii* so4 and *Coniochaeta* sp. 2T2.1. In terms of gene expression, *S. paramultivorum* w15 was not heavily affected by shaking speed, as the trends in the patterns (i.e., *ftsA, ftsZ* and *rpoD*) were similar between 180 and 60 rpm. However, a clear trend of early growth onset, concomitant with a fast stress response (high expression of the *dps* gene), can be seen from Additional file 2: Fig. S3a. Rapid growth accompanied by stress responses is known for other systems, and possibly indicates quite ‘healthy’ and fast responses by the investigated organisms to local conditions. Interestingly, *S. paramultivorum* w15 may have faced less (starvation) stress at 60 versus 180 rpm, as indicated by the significantly lower *dps* gene expression at late stage at low shaking speed.

### *C. freundii* so4

As a facultatively anaerobic bacterium, *C. freundii* so4 may have—to a considerable extent—used routes for energy generation different from aerobic respiration. Thus, it may have, to varying extents, switched from aerobic to flexible (aerobic/microaerophilic/anaerobic) metabolism, in particular at 60 rpm (Additional file 2: Fig. S3). Its continued (slow) growth, even at late stage at 60 rpm, could be due to this flexible energy-generating mechanism, as all metabolisms other than aerobic are, as one understands, less energy-efficient. We did not do a full metabolic study of all possible processes, as this is beyond the scope of this study. Moreover, the high expression levels of genes involved in stress responses (*elaB*, *uspB*, *bssR*, *bssS*; Additional file 2: Fig. S8) at both shaking speeds indicated that *C. freundii* so4 may live in the consortium in an “alerted” (potentially ‘shielded’) fashion. In such a state, it may be optimally tuned to respond to stress, with the connotation that the lower expression levels of stress-related genes at 60 rpm suggested that less stressful conditions reigned *C. freundii* so4 behavior at 60 rpm. The putative role of the products of the *bssR* and *bssS* genes was derived from their likely involvement in repression of biofilm formation, as observed in continuous-flow chambers with minimal glucose medium [22]. Thus, we presumed the lower expression of these two genes at late stage at 60 rpm may have resulted in a higher level of biofilm biomass (higher cell aggregation levels) at 60 rpm. This is consistent with a previous tenet [21].

### *Coniochaeta* sp. 2T2.1

The expression patterns of the selected genes encoding cell shape (*egf1*) and adhesion glycoproteins (KOG1437) supported the previous finding that *Coniochaeta* sp. 2T2.1, in response to the conditions imposed on the cultures by modulating shaking speed (high, turbulent versus low, laminar to almost still), switched between the multicellular hyphal and the unicellular yeast forms. We hypothesized that the observed modulation of the expression of two *egf1* genes (encoding “enhanced filamentous growth protein” EGF1) was at the basis of such morphological adaptations (Additional file 2: Fig. S3). In concrete terms, the lower shaking speed likely favored mycelial growth, whereas the higher shaking speed repressed it. The trigger for this cell type switch may also be associated with nutritional (including oxygen) and/or condition changes, similar to what has been found in other dimorphic fungi [16]. *Coniochaeta* sp. 2T2.1 is postulated to have a strict aerobic growth mode, and it is likely that, at 60 rpm, heterogeneity of oxygen supply modulates multicellular hyphal growth, in which, at lower oxygen level, *Coniochaeta* sp. 2T2.1 is predicted to mainly grow as hyphae extending to their surroundings for more oxygen, just like in natural environments. Outgrowth of hyphae to achieve higher oxygen levels can facilitate diffusion and transport of compounds, and promote nutritive absorption. For instance, the hyphae of the “yeast” *Candida albicans* have been reported to extend towards oxygen-rich directions when exposed to gradients of oxygen [24].

The differential behavior of each of the consortial organisms, especially *Coniochaeta* sp. 2T2.1, might also be consistent with the significantly higher WS weight loss at 60 rpm as compared to 180 rpm (Fig. 1). One might speculate that—at 60 rpm—the adhering fungal hyphae benefit consortial members to attach to the surface of WS, thus enhancing WS degradation efficiency. In addition, hyphae may provide more sites for excreting compounds (enzymes), and so, at similar gene expression levels, higher amounts of protein might be released in by hyphae than by yeast-like cells.

Not only the behavior of the three members in the consortium differed between two shaking conditions, but the expression of CAZy families and enzymatic synergism were also affected by the shaking speed as discussed hereunder.

### Putative complementary enzymatic mechanisms leading to division of labor

Examination of the highly expressed genes of *Coniochaeta* sp. 2T2.1 (Fig. 3) revealed a major role of the organism in lignocellulolytic enzyme production. In particular, the unique genes encoding enzymes potentially involved in lignin (AA1_3, AA3_3), cellulose (AA9, AA16, GH7), arabinoxylan backbone (GH11) and side chain (CE5, GH62, GH27) degradations stood out as being uniquely fungal, as those genes were absent from the genomes of both bacteria [5]. Thus division of labor within the consortium implies a major involvement of *Coniochaeta* sp. 2T2.1 in the aforementioned metabolic routes. Such activities might influence the access of all consortial members to the substrate, as previously indicated [4].

The potentially enhanced substrate access also implies the WS hemicellulose moieties. These can be divided into three classes (xylans, mannans and xyloglucans) on the basis of their structure [25]. The highly (and stably) expressed genes encoding proteins of CAZy classes GH43, CE1, GH2, GH92, GH95 and GH29 in *S. paramultivorum* w15 pointed at the occupancy by strain w15 of key niches at both shaking speeds with basis in the hemicellulose fractions arabinoxylan (GH43, CE1), galactoglucomannan (GH2, GH92) and xyloglucan (GH95, GH29) (Fig. 2). In contrast, *Coniochaeta* sp. 2T2.1 appeared to more strongly prefer arabinoxylan-defined niches (Fig. 3). The ‘sharing of’—or competition for—arabinoxylan-defined niches between *S. paramultivorum* w15 and *Coniochaeta* sp. 2T2.1 may have resulted in the observed repression of genes encoding arabinoxylan-attacking proteins in *Coniochaeta* sp. 2T2.1 at early stage at 180 rpm. However, this phenomenon was also controlled by the shaking conditions, as such repression was not found at 60 rpm, in early growth stage (Fig. 3). On the other hand, complementary enzymatic mechanisms could be taking place given the complementary CAZy-protein encoding genes in each genome (Table 2).

Consistent with our previous observations, we here describe the role of *C. freundii* so4 in WS degradation as being largely “secondary” [5]. However, a key role of dealing with oxygen uptake and possible competition (Additional file 2: Fig. S3c) by switching from aerobic to flexible (aerobic/microaerophilic/anaerobic) respiration at 60 rpm, next to one in detoxification (using different strategies under two shaking speeds), can be cogitated (Additional file 2: Fig. S7). The high expression levels of stress-response associated genes in *C. freundii* so4 also indicated that stress conditions (starvation, competition for niches) were major drivers of gene expression. Next to nutrient competition, such stressors can be of any other (abiotic or biotic) nature. A further study of the stress alleviation function (i.e. clean-up of toxicity) of *C. freundii* so4 would be of great eco-enzymatic interest.

### Additional potential competition for niches between *Coniochaeta* sp. 2T2.1 and *S. paramultivorum* w15 at 180 rpm

The low expression levels of *Coniochaeta* sp. 2T2.1 genes involved in hemicellulose and cellulose degradation at early to middle stage at 180 rpm (Fig. 3) indicated potential gene repression by (any of) the bacterial consortium member(s). The specific repression of genes encoding enzymes involved in arabinofuranoxylan (CAZy classes GH11, CE1, GH62, GH51, GH127, GH43, GH93, GH27 and GH3) and cellulose (AA3_2, GH71, GH7 and GH12) degradation was indeed consistent with that reported previously [4]. Thus, as available niches determine the activities and interactions, (hemi) cellulose-determined niches of the WS may have been mainly occupied by *S. paramultivorum* w15 (Fig. 2). The fact that this niche occupancy occurred early at 180 rpm is in line with the observed very fast response of *S. paramultivorum* w15 to the WS [21]. It is also supported by the tenet that the presence of multiple 16S rRNA operons offers ample possibilities of ‘kick starting’ rapid metabolism upon perception of such ecological opportunities [26]. Another facet here is the possibility that bacterial enzymes may be physically different, with enzymes best ‘fitting’ the substrate allowing more efficient activities, ultimately raising the number of key enzymes attacking hemi(cellulose) in the system.

### Metabolic synergism may be promoted by low shaking speed

Several *Coniochaeta* sp. 2T2.1-derived genes encoding biodegradative enzymes (CAZy classes GH5, GH7, AA9, GH10, GH11, CE1, CE5 and GH43) were repressed at early stage at 180 rpm (Fig. 3), but clearly not at 60 rpm (Fig. 3). This resulted in the fungal degradation activities beginning earlier at 60 than at 180 rpm. Thus, ligninolytic activities of *Coniochaeta* sp. 2T2.1 may have been promoted by low shaking speed simply because the relevant substrate structures became exposed at an earlier stage. It has been reported that many ligninolytic *Basidiomycetous* fungi will mainly secrete the types of oxidative lytic enzymes that commonly attack lignin under nutrient-limited conditions [27]. Here, we found a significantly higher expression of genes encoding enzymes involved in lignin transformation (CAZy classes AA3, AA3_3 and AA3_2) at 60 rpm compared to 180 rpm (late stage; Fig. 3). Similarly, *S. paramultivorum* w15 might have an important role in lignin transformation at lower shaking speed (Fig. 2). In contrast, we postulate the role of *C. freundii* so4 to be that of a ‘consumer’, as revealed by its expression of genes encoding CAZy family GH1 and GH3 proteins.

Next to the enzymatic synergism, complementary functions of each member of the consortium might facilitate microbial coexistence.

### *S. paramultivorum* w15

Among the top-200 of highly expressed genes, several genes, in particular *fixA*, *fixB* and *ribE*, with roles in vitamin B2 metabolism were found (Fig. 4b, Fig. 5), with clear trends in their dynamics. Gene *ribE* catalyzes the dismutation of two molecules of 6,7-dimethyl-8-ribityllumazine, resulting in the formation of riboflavin. One can assume that, at early stage, with sufficient vitamin B2 in the media, the expression of these synthesis genes is low, while at later stages it depends, for each organism, on its respective growth/metabolic rate,  i.e. maintaining higher cell density also requires more involvement of these gene. Our data also raise the possibility that *S. paramultivorum* w15 is involved in the production of B2 vitamins at early stages (across the two shaking speeds), and in late stage at 180 rpm. At 60 rpm, a later involvement (late stage) of *C. freundii* so4 is possible, pointing to a putative role switch (Fig. 5). Vitamin B2 (riboflavin), a precursor of the coenzymes flavin mononucleotide and flavin adenine dinucleotide, is essential for life, as it is involved in key cellular redox reactions, such as the conversion of vitamins B6 and B9 into their active forms, as well as the mobilization of iron [28]. The fact that we found the genes involved in vitamin B2 generation to be highly expressed early on at 60 rpm may indicate a high requirement of such oxidoreductive systems in this early phase under potentially high levels of (oxygen) stress. We assumed that the initial key role of *S. paramultivorum* w15 was later supported by *C. freundii* so4 and *Coniochaeta* sp. 2T2.1 (Fig. 5). Further confirmation of the trends and the cross talk in vitamin B2 production in our consortium will constitute a very interesting follow-up of the current study.

### *C. freundii* so4

*C. freundii* so4 may be involved in clearing dead cells from the system by upregulating the *cvpA* gene (implicated in disruption of cell membranes) at late stage, particularly at 60 rpm. The gene *cvpA* encoded protein has been linked to colicin V secretion in *Escherichia coli*, and a role in response to stress stimuli in diverse bacterial species was also suggested [29]. Reduction of adherence processes in the culture might ensue, which is consistent with the earlier finding of less adhesion in tripartite than in fungal monocultures at 60 rpm [21]. This is in line with the higher expression of gene *bhsA* at late stage at 60 rpm (Additional file 2: Fig. S7), which can decrease cell surface hydrophobicity and cell aggregation, and the significantly higher expression of *mcbA,* as deletion of *mcbA* increased biofilm formation of in *E. coli* LB medium [23].

### *Coniochaeta* sp. 2T2.1

The observation that more adhesins are induced at 60 than at 180 rpm was remarkable. Such proteins containing fasciclin domains, which are predicted to be located in extracellular space [30], and are reported to be associated with cell adhesion functions. Thus the observed higher expression of genes encoding fasciclin and adhesion glycoproteins in *Coniochaeta* sp. 2T2.1 at 60 rpm (Additional file 2: Fig. S3) was consistent with the observed higher cell aggregation levels at this shaking speed. Perhaps, the hyphal form also contributes to the adhesion at 60 rpm. For *C. albicans,* it has been shown that hyphae are more hydrophobic than yeast-like cells, as the main adherent proteins of germ tubes arising from cells to initiate hyphal growth are hydrophobic [31]. Cell surface hydrophobicity thus plays a crucial role in adherence processes [32]. Thus, at 60 rpm, *Coniochaeta* sp. 2T2.1 may benefit from its formation of multicellular hyphae and extended cell surfaces, which can produce attachment sites for glycoproteins. This promotes the high cell aggregation levels at low (versus high) shaking speed.

### Examination of selected expressed genes

To shed light on the nature of the *Coniochaeta* sp. 2T2.1 messengers detected by us, we examined the phylogenetic placement of the two genes encoding bystins and eight encoding adhesion glycoproteins. We demonstrate that the former two genes cluster with a suite of bystin genes from other fungi (Additional file 2: Fig. S9a), whereas the eight adhesion glycoprotein genes cluster into four clades of adhesion glycoproteins belonging to *C. ligniaria*, *Fusarium oxysporum*, *Coniochaeta* sp. PMI 546, and *Coniochaeta* sp. 2T2.1 itself. This is shown in Additional file 2: Fig. S9b. A tight clustering is apparent, with amino acid sequence similarities of 92–98% (Additional file 2: Table S2). Given that the latter eight genes were found to be distributed along the whole genome, being localized on different chromosomes, we hypothesized that their origins were not strongly linked, and so the resulting glycoproteins might have different structure, different binding sites, and probably different substrate binding mechanisms. Thus, a variety of ecological ‘choices’ may allow *Coniochaeta* sp. 2T2.1 to form adhesion complexes under various conditions, increasing its chance of establishment and survival via better attachment to emerging substrates. The four clusters we defined appear to support this assumption.

### Degradation of arabinogalactan-proteins/proteoglycans was upregulated only at 180 rpm

The role of genes involved in fungal/bacterial cell wall decomposition modulations was remarkable. First, polysaccharides (β-glucans) and glycoproteins have been reported to play roles in the adhesion of bacteria and fungi to substrates [33, 34], and hence their removal might affect adherence. Here, the higher expression of *Coniochaeta* sp. 2T2.1 genes involved in arabinogalactan–protein degradation (GH79) at 180 rpm compared to 60 rpm could result in lower cell agglomeration rates (Additional file 2: Fig. S4). As reported by Jiménez et al. [4], GH79-like enzymes may attack arabinogalactan-proteins/proteoglycans in plant and bacterial cell walls [35]. The two bacteria lack genes for GH79 family proteins, but proteins produced by genes encoding peptidoglycan lyase (GH73) may have similar effects on bacterial cell walls. The expression dynamics of genes for GH73 (and GH23) proteins in *C. freundii* so4 showed other trends, with both genes being highly expressed at late stage at 60 rpm. A possible involvement in cleaning of accumulated dead cells is cogitated (Additional file 2: Fig. S4), as proteins in both GH23 and GH73 families share a typical lysozyme-like α/β fold, cleave the β-1,4-glycosidic linkage between N-acetylglucosaminyl (NAG) and N-acetylmuramyl (NAM) moieties in the carbohydrate backbone of bacterial peptidoglycans [36]. *C. freundii* so4 has previously been reported to consume putrescine, which is produced by the breakdown of amino acids in living and dead organisms and is toxic in large doses [5].

### Fungal form changes and adhesion drive interactions

Glycans or derivatives (β-glucans) are essential components of the matrix in *C. albicans* biofilms [37], and so one may surmise this is also the case in *Coniochaeta* sp. 2T2.1 biofilms. Since, in our oligospecies consortia, the two bacteria are capable of producing various enzymes attacking glycans [5], the adhesive/agglomerative properties of *Coniochaeta* sp. 2T2.1 may have been affected in the cultures by enzyme-driven mechanisms. Moreover, the hyphal form may also modulate (positive) interactions between bacteria and fungi, as reported previously [37–39], with hyphae serving as “highways” for bacteria to move to their niches, colonizing accessible sites of the WS.

As part of the nature of transcriptomics studies, the sequence-based annotation of genes can be 'tricky', as the binding pockets and amino acid sequences of orthologs can be very similar, but work on distinct carbohydrates with (to some extent) similar but different stereochemistry. However, based on the different levels of regulation found in this study, we feel the aforementioned hypotheses have sufficient strength to be put forward.

## Conclusions

Here, we explored the impact of two bacterial strains and one fungus, in terms of degradation of the WS (hemi)cellulose and modifying lignin moieties, in relation to local conditions as defined by shaking speed. We posit that *S. paramultivorum* w15 is involved in the degradation of mainly hemicellulose (high expression of diverse hemicellulases) and vitamin B2 production, and *C. freundii* so4 in the degradation of oligosaccharides and/or sugar dimers, next to detoxification processes, with both strains consuming the sugar monomers (e.g., glucose and xylose) resulting from the degradation process. We further provide evidence for the tenet that *Coniochaeta* sp. 2T2.1 may be most strongly involved in cellulose and xylan (at early stages), next to lignin degradation processes (at later stages). Considering the effects of conditions, we show the physical life forms and appearances of the organisms, in particular the fungal partner, to be determinative for the biodegradative processes studied, and hence for the associated gene expression patterns. Modulation of shaking speed theoretically results in contrasting conditions with respect to the degree of mixing, heterogeneity, adherence the substrate, physical appearance and distribution of compounds like molecular oxygen (high, turbulent, high degree of mixing, versus low, almost still). This study thus enhances the eco-enzymological understanding of the degradation of WS by a tripartite microbial consortium, revealing the synergism between the members and alternative functional roles that allow their coexistence in this culture system. The consortium studied here, representing three members derived from a natural microbial community, may serve as a model consortium for further evolutionary ecological studies, or a novel platform for consolidated bioprocesses in conjunction with metabolic engineering and genome-scale metabolic models. The key enzymes (CAZy classes GH10, GH11, CE1, CE5 and GH43) relevant for the initial stages of wheat straw degradation, converting the macromolecules (i.e., (hemi)cellulose, lignin and pectin) to smaller molecules, could be employed in strategies to produce compounds for use in the food, textile and pharmaceutical industries. Furthermore, the enzymes pinpointed in this study provide molecular 'goldmines' for further characterization and expression, potentially resulting in production for commercial applications. Further work unraveling the interactions at the protein level would be helpful for assessing the reliability of the functions assigned to the expressed genes assigned herein.

## Materials and methods

### Strains, experimental setup and tripartite microbial consortium culture

Two bacteria, *Citrobacter freundii* so4 (DSM106340T) and *Sphingobacterium paramultivorum* w15 (DSM 106342), and one fungus, *Coniochaeta* sp. 2T2.1 (NRRL Y-64006), were used as members of the constructed microbial consortium. To set up the experiment, a liquid medium was prepared using WS as the sole carbon source. 0.3 g of washed WS (1%, *w*/*v*) were placed in 30 mL mineral medium (7 g/L Na_2_HPO_4_·2H_2_O; 2 g/L K_2_HPO_4_; 1 g/L (NH_4_)_2_SO_4_; 0.1 g/L Ca(NO_3_)_2_·4H_2_O; 0.2 g/L MgCl_2_·6H_2_O g/L) in 100-ml Erlenmeyer flasks. The media were supplemented with 30 μL vitamin solution (0.1 g Ca-pantothenate, 0.1 g cyanocobalamin, 0.1 g nicotinic acid, 0.1 g pyridoxal, 0.1 g riboflavin, 0.1 g thiamin, 0.01 g biotin, 0.1 g folic acid; H_2_O 1 L) and 30 μL trace metal solution (2.5 g/L EDTA; 1.5 g/L FeSO_4_·7H_2_O; 0.025 g/L CoCl_2_; 0.025 g/L ZnSO_4_·7H_2_O; 0.015 g/L MnCl_2_; 0.015 g/L NaMoO_4_·2H_2_O; 0.01 g/L NiCl_2_; 0.02 g/L H_3_BO_3_; 0.005 g/L CuCl_2_) as previously described [6]. The experiments were performed at 28 °C, with initial pH 6.2, under two shaking speeds (180 and 60 rpm) in two comparable rotary shakers (INFORS HT, Bottmingen, Switzerland). The initial cell density of each strain was about 5–6 log cells per mL. Estimation of WS degradation was done by using WS weight loss, whereas microbial culture and growth measurements were done by colony forming unit (CFU) counting as described previously [21].

### Microbial RNA extraction and sequencing

At each time point (1 day, 5 and 13 d), three flasks from each shaking speed were used to harvest the cells and extract total RNA. The flasks were vigorously shaken for 10 s, and 15 ml liquid was transferred to 15-ml tubes sitting on ice. The tubes were then centrifuged at 4 ℃ at 7189xg, for sufficient time to pellet all cells (up to 10 min), after which the supernatant was carefully discarded. Then, the tubes were centrifuged and inverted again, to remove liquid totally. RNA extractions were conducted right after harvesting the samples, following the instructions of the RNeasy Kit (Qiagen, GmbH, Germany). RNA quality was checked using agarose gel electrophoresis (for physical observation) and spectrophotometry using the absorbance ratios 260/280 nm. Poly-A tail enrichment was used for fungal RNA using NEBNext Poly(A) Magnetic Isolation Module (New England Biolabs, Frankfurt am Main, Germany) and rRNA depletion for bacterial RNA following the Pan-Pro Ribo-Pool protocol. Sequencing on Illumina NextSeq500/550 (2 × 150 bp) was performed at LGC Genomics (Berlin, Germany).

### Data analysis

Raw data with adapter clipped reads were cleaned using Fastp software (version 0.21.0, [40]). Reads with any Ns or those shorter than 25 bp were removed. SortMeRna (version 4.2.0, [41]) was used to further sort out ribosomal RNA reads. The remaining mRNA reads were mapped against the reference genome of each strain [5, 16]. Hisat2 (version 2.2.1, [42]) was used to map fungal mRNA reads under very-sensitive mode; Bowtie2 (version 2.4.4, [43]) was used for mapping bacterial sequences under very-sensitive-local mode. FeatureCounts (version 2.0.1, [44]) was used to generate the raw gene read counts file using paired-read models. Principal components analysis (PCA) was used to visualize the triplicates using RStudio (version 1.4.1106, [45]; R version 4.0.4, [46]) software, which was also used to calculate the Transcripts Per Kilobase Million (TPM) values, and TPM values were used to compare gene expression within conditions. To get an overview of expressed genes, the mean TPM values were calculated for each sample, after which genes of the top-200 highest mean TPM values were selected as “highly expressed genes” from each sample. These genes were further annotated by KEGG and dbCAN [47, 48]. TPM normalized read counts of each gene belonging to the same CAZy family at each condition and at each time point were summarized and used for visualization. Gene TPM values were grouped and labeled as: (1) high expression level (TPM above 1000), (2) intermediate expression level (TPM between 100 and 1000), and low expression level (TPM below 100). DESeq2 (version 1.30.1, [49]) was subsequently used to determine differentially expressed genes between pairs of conditions. Genes were annotated using the KEGG database using GhostKOALA. In addition, annotation of Carbohydrate-Active Enzymes (CAZy) and the non-catalytic carbohydrate-binding modules (CBMs) was performed using dbCAN [47, 48]; HMMER output (E-value < 1e^−15^, coverage > 0.35) was firstly used, then Diamond and Hotpep searches against the CAZy database were also considered. Signal peptide was checked using results from dbCAN.

### Selection of genes with potential relevance for consortial growth on WS

To examine the mechanisms potentially underlying the growth-associated and agglomeration/adhesion-like behaviors observed, we monitored the expression of several ‘proxies’ genes for these functions. For the two bacteria, we selected the ‘growth’ indicator genes *ftsA* and *ftsZ* (involved in assembly of the Z ring in bacterial cell division; [50]), as well as exponential growth indicator gene *rpoD* (encoding the primary sigma factor RpoD, key during exponential growth; [51]) and stationary phase-induced genes *bolA* (encoding the general stress response regulator; [52]) and *elaB* (encoding the ElaB protein—protecting cells against oxidative stress; [53]). Due to the absence of these two genes (*bolA, elaB*) in *S. paramultivorum* w15, a *dps-*like gene (encoding a starvation-inducible DNA-binding protein; [54]) was examined. Moreover, for the facultatively anaerobic *C. freundii* so4, both aerobic respiration (*arcA*, [55]) and anaerobic regulatory (*fnr*, [55]) related genes were examined*.* For *Coniochaeta* sp. 2T2.1, genes *egf1* encoding “enhanced filamentous growth protein 1” (EFG1—potentially involved in hyphal formation; [56]) were taken into account. In addition, two genes encoding so-called “cell adhesion complex” proteins (bystin; KOG3871) and eight genes predicted to encode the adhesion glycoprotein fasciclin (KOG1437) were examined.

## Supplementary Information

**Additional file 1: Table S1.** Transcriptomic raw data (libraries names and raw counts in each library).

**Additional file 2: Table S2.** Distribution of adhesion related genes of *Coniochaeta* sp. 2T2.1. **Figure S1.** Physical appearance of (a and d) bacterial–fungal consortium (Coniochaeta sp. 2T2.1, Sphingobacterium paramultivorum w15 and Citrobacter freundii so4) and (b and c) monoculture of Coniochaeta sp. 2T2.1 at 60 rpm (a and b) and 180 (c and d) rpm using wheat straw as the sole of carbon and energy source. **Figure S2.** Principal components analysis (PCA) of the expression profile of (a) *Citrobacter freundii* so4, (b) *Sphingobacterium paramultivorum* w15 and (c) *Coniochaeta* sp. 2T2.1. **Figure S3.** Growth indicator genes dynamics of (a) *Sphingobacterium paramultivorum* w15 and (b) *Citrobacter freundii* so4; (c) respiration regulating genes of *Citrobacter freundii* so4; (d) hyphal and (e) adhesion genes of *Coniochaeta* sp. 2T2.1 at 180 rpm (black) and 60 rpm (grey). **Figure S4.** Expression dynamics of peptidoglycan related genes in (a) *Citrobacter freundii* so4 and (b) *Sphingobacterium paramultivorum* w15. **Figure S5.** Expression dynamics of proteoglycans-attacking related genes expressed at 180 rpm (black) and 60 rpm (grey) in *Coniochaeta* sp. 2T2.1. **Figure S6.** Reactive oxygen species (ROS) protection genes in *Sphingobacterium paramultivorum* w15. **Figure S7.** Expression dynamics of detoxification related genes in *Citrobacter freundii* so4. **Figure S8.** Expression dynamics of biofilm and stress related genes in *Citrobacter freundii* so4. **Figure S9.** Phylogenetic tree of (a) bystin and (b) glycoproteins of *Coniochaeta* sp. 2T2.1.

**Additional file 3: Table S3.** CAZy family gene expressions data of *Citrobacter freundii* so4, *Sphingobacterium paramultivorum* w15 and *Coniochaeta* sp. 2T2.1 at different shaking speed over time.

## Data Availability

The data sets supporting the findings of this study are included as Additional files 1 to 3. The transcriptome data were deposited at DDBJ/EMBL/GenBank databases under the following SRA accessions BioProject ID PRJNA870051.

## Abbreviations

LCB: Lignocellulosic biomass
LPMO’s: Lytic polysaccharide monooxygenases
WS: Wheat straw
PCA: Principal components analysis
TPM: Transcripts per kilobase million
LCMO: Lytic cellulose monooxygenase
AAO: Aryl alcohol oxidase

## References

[1] Van Dyk J, Pletschke B (2012). A review of lignocellulose bioconversion using enzymatic hydrolysis and synergistic cooperation between enzymes—factors affecting enzymes, conversion and synergy. Biotechnol Adv.

[2] Zhang YHP, Lynd LR (2004). Toward an aggregated understanding of enzymatic hydrolysis of cellulose: noncomplexed cellulase systems. Biotechnol Bioeng.

[3] Jiménez DJ, De Chaib MM, Salles JF (2018). Temporal expression dynamics of plant biomass-degrading enzymes by a synthetic bacterial consortium growing on sugarcane bagasse. Front Microbiol.

[4] Jiménez DJ, Wang Y, Chaib de Mares M, Cortes-Tolalpa L, Mertens JA, Hector RE, Lin J, Johnson J, Lipzen A, Barry K, Mondo SJ, Grigoriev IV, Nichols NN, van Elsas JD (2020). Defining the eco-enzymological role of the fungal strain Coniochaeta sp. 2T2.1 in a tripartite lignocellulolytic microbial consortium. FEMS Microbiol Ecol.

[5] Cortes-Tolalpa L, Wang Y, Salles JF, van Elsas JD (2020). Comparative genome analysis of the lignocellulose degrading bacteria *Citrobacter freundii* so4 and *Sphingobacterium multivorum* w15. Front Microbial.

[6] Jiménez DJ, Korenblum E, van Elsas JD (2014). Novel multispecies microbial consortia involved in lignocellulose and 5-hydroxymethylfurfural bioconversion. Appl Microbiol Biotechnol.

[7] Cortes-Tolalpa L, Jiménez DJ, de Lima Brossi MJ, Salles JF, van Elsas JD (2016). Different inocula produce distinctive microbial consortia with similar lignocellulose degradation capacity. Appl Microbiol Biotechnol.

[8] Díaz-García L, Chaparro D, Jiménez H, Gómez-Ramírez LF, Bernal AJ, Burbano-Erazo E, Jiménez DJ (2021). Top-down enrichment strategy to co-cultivate lactic acid and lignocellulolytic bacteria from the megathyrsus maximus phyllosphere. Front Microbiol.

[9] Díaz-García L, Huang S, Spröer C, Sierra-Ramírez R, Bunk B, Overmann J, Jiménez DJ (2021). Dilution-to-stimulation/extinction method: a combination enrichment strategy to develop a minimal and versatile lignocellulolytic bacterial consortium. Appl Environ Microbiol.

[10] Cortes-Tolalpa L, Salles JF, van Elsas JD (2017). Bacterial synergism in lignocellulose biomass degradation–complementary roles of degraders as influenced by complexity of the carbon source. Front Microbial.

[11] Puentes-Téllez PE, Falcao JS (2018). Construction of effective minimal active microbial consortia for lignocellulose degradation. Microb Ecol.

[12] Shahab RL, Brethauer S, Davev MP, Smith AG, Vignolini S, Luterbacher JS, Studer MH (2020). A heterogeneous microbial consortium producing short-chain fatty acids from lignocellulose. Science.

[13] Jiménez DJ, Maruthamuthu M, Elsas JD (2015). Metasecretome analysis of a lignocellulolytic microbial consortium grown on wheat straw, xylan and xylose. Biotechnol Biofuels.

[14] Photphisutthiphong Y, Vatanyoopaisarn S (2019). *Dyadobacter* and *Sphingobacterium* isolated from herbivore manure in Thailand and their cellulolytic activity in various organic waste substrates. Agric Nat Resour.

[15] Chandra R, Abhishek A, Sankhwar M (2011). Bacterial decolorization and detoxification of black liquor from rayon grade pulp manufacturing paper industry and detection of their metabolic products. Bioresour Technol.

[16] Mondo SJ, Jiménez DJ, Hector RE, Lipzen A, Yan M, LaButti K, Barry K, van Elsas JD, Grigoriev IV, Nichols NN (2019). Genome expansion by allopolyploidization in the fungal strain Coniochaeta 2T2.1 and its exceptional lignocellulolytic machinery. Biotechnol Biofuels.

[17] Ravindran A, Adav SS, Sze SK (2012). Characterization of extracellular lignocellulolytic enzymes of *Coniochaeta* sp. during corn stover bioconversion. Process Biochem.

[18] Simmons CW, Reddy AP, D’haeseleer P, Khudyakov J, Billis K, Pati A, Simmons BA, Singer SW, Thelen MP, VanderGheynst JS (2014). Metatranscriptomic analysis of lignocellulolytic microbial communities involved in high-solids decomposition of rice straw. Biotechnol Biofuels.

[19] Serrano-Gamboa JG, Fócil-Espinosa CA, Cabello-Yeves PJ, Coutinho FH, Rojas-Herrera RA, Sánchez-González MN (2022). Metatranscriptome profiling of a specialized microbial consortium during the degradation of nixtamalized maize pericarp. Microbiol Spectr.

[20] Mello BL, Alessi AM, Riaño-Pachón DM, deAzevedo ER, Guimarães FEG, Santo MCE, McQueen-Mason S, Bruce NC, Polikarpov I (2017). Targeted metatranscriptomics of compost-derived consortia reveals a GH11 exerting an unusual exo-1,4-β-xylanase activity. Biotechnol Biofuels.

[21] Wang Y, Elzenga T, van Elsas JD (2021). Effect of culture conditions on the performance of lignocellulose-degrading synthetic microbial consortia. Appl Microbiol Biotechnol.

[22] Domka J, Lee J, Wood TK (2006). liH (BssR) and YceP (BssS) regulate *Escherichia coli* K-12 biofilm formation by influencing cell signaling. Appl Environ Microbiol.

[23] Zhang XS, García-Contreras R, Wood T (2008). *Escherichia coli* transcription factor YncC (McbR) regulates colanic acid and biofilm formation by repressing expression of periplasmic protein YbiM (McbA). ISME J.

[24] Aoki S, Ito-Kuwa S, Nakamura K, Vidotto V, Takeo K (1998). Oxygen as a possible tropic factor in hyphal growth of *Candida albicans*. Mycoscience.

[25] Frassoldati A, Ranzi E, Frassoldati A (2019). Modeling of thermochemical conversion of biomasses. reference module in chemistry, molecular sciences and chemical engineering.

[26] Wang Y, Brons JK, van Elsas JD (2021). Considerations on the identity and diversity of organisms affiliated with *Sphingobacterium multivorum*—proposal for a new species. Sphingobacterium paramultivorum Microorganisms.

[27] Saparrat MCN, Martínez MJ, Tournier HA, Cabello MN, Arambarri AM (2000). Production of ligninolytic enzymes by *Fusarium solani* strains isolated from different substrata. World J Microbiol Biotechnol.

[28] Averianova LA, Balabanova LA, Son OM, Podvolotskaya AB, Tekutyeva LA (2020). Production of vitamin B2 (riboflavin) by microorganisms: an overview. Front bioeng biotechnol.

[29] Warr AR, Giorgio RT, Waldor MK (2020). Genetic analysis of the role of the conserved inner membrane protein CvpA in EHEC resistance to deoxycholate. J Bacteriol.

[30] Liu E, MacMillan CP, Shafee T, Ma Y, Ratcliffe J, van de Meene A, Bacic A, Humphries J, Johnson KL (2020). Fasciclin-like arabinogalactan-protein 16 (FLA16) is required for stem development in *Arabidopsis*. Front Plant Sci.

[31] Tronchin G, Bouchara JP, Robert R, Senet JM (1988). Adherence of *Candida albicans* germ tubes to plastic: ultrastructural and molecular studies of fibrillar adhesins. Infect Immun.

[32] Tronchin G, Pihet M, Lopes-Bezerra LM, Bouchara JP (2008). Adherence mechanisms in human pathogenic fungi. Med Mycol J.

[33] Epstein L, Nicholson R, Smith AM, Callow JA (2006). Adhesion and adhesives of fungi and oomycetes. Biological adhesives.

[34] Lipke PN (2018). What we do not know about fungal cell adhesion molecules. J Fungi.

[35] Knoch E, Dilokpimol A, Geshi N (2014). Arabinogalactan proteins: focus on carbohydrate active enzymes. Front Plant Sci.

[36] Lipski A, Hervé M, Lombard V, Nurizzo D, Mengin-Lecreulx D, Bourne Y, Vincent F (2015). Structural and biochemical characterization of the β-N-acetylglucosaminidase from *Thermotoga Maritima*: toward rationalization of mechanistic knowledge in the GH73 family. Glycobiology.

[37] Warmink JA, Nazir R, van Elsas JD (2009). Universal and species-specific bacterial “fungiphiles” in the mycospheres of different basidiomycetous fungi. Environ Microbiol.

[38] Trifonova R, Postma J, van Elsas JD (2009). Interactions of plant-beneficial bacteria with the ascomycete *Coniochaeta ligniaria*. J Appl Microbiol.

[39] Simon A, Bindschedler S, Job D, Wick LY, Filippidou S, Kooli WM, Verrecchia EP, Junier P (2015). Exploiting the fungal highway: development of a novel tool for the in situ isolation of bacteria migrating along fungal mycelium. FEMS Microbiol Ecol.

[40] Chen S, Zhou Y, Chen Y, Gu J (2018). fastp: an ultra-fast all-in-one FASTQ preprocessor. Bioinformatics.

[41] Kopylova E, Noe L, Touzet H (2012). SortMeRNA: fast and accurate filtering of ribosomal RNAs in metatranscriptomic data. Bioinformatics.

[42] Kim D, Langmead B, Salzberg SL (2015). HISAT: a fast spliced aligner with low memory requirements. Nat Methods.

[43] Langmead B, Salzberg S (2012). Fast gapped-read alignment with Bowtie 2. Nat Methods.

[44] Liao Y, Smyth GK, Shi W (2014). featureCounts: an efficient general purpose program for assigning sequence reads to genomic features. Bioinformatics.

[45] RStudio Team. RStudio: integrated development environment for R. RStudio, PBC, Boston, MA. 2021; URL http://www.rstudio.com/

[46] R Core Team (2021). R: A language and environment for statistical computing.

[47] Yin Y, Mao X, Yang J, Chen X, Mao F, Xu Y (2012). dbCAN: a web resource for automated carbohydrate-active enzyme annotation. Nucleic Acids Res.

[48] Zhang H, Yohe T, Huang L, Entwistle S, Wu P, Yang Z, Busk P, Xu Y, Yin Y (2018). dbCAN2: a meta server for automated carbohydrate-active enzyme annotation. Nucleic Acids Res.

[49] Love MI, Huber W, Anders S (2014). Moderated estimation of fold change and dispersion for RNA-seq data with DESeq2. Genome Biol.

[50] Loose M, Mitchison T (2014). The bacterial cell division proteins FtsA and FtsZ self-organize into dynamic cytoskeletal patterns. Nat Cell Biol.

[51] Wösten MM (1998). Eubacterial sigma-factors. FEMS Microbiol Rev.

[52] Santos JM, Freire P, Vicente M, Arraiano CM (1999). The stationary-phase morphogene bolA from Escherichia coli is induced by stress during early stages of growth. Mol Microbiol.

[53] Guo Y, Li Y, Zhan W, Wood TK, Wang X (2019). Resistance to oxidative stress by inner membrane protein ElaB is regulated by OxyR and RpoS. Microb Biotechnol.

[54] Calhoun LN, Kwon YM (2011). Structure, function and regulation of the DNA-binding protein Dps and its role in acid and oxidative stress resistance in *Escherichia coli*: a review. J Appl Microbiol.

[55] Rolfe MD, Ter Beek A, Graham AI, Trotter EW, Asif HM, Sanguinetti G, de Mattos JT, Poole RK, Green J (2011). Transcript profiling and inference of *Escherichia coli* K-12 ArcA activity across the range of physiologically relevant oxygen concentrations. J Biol Chem.

[56] Riggle PJ, Andrutis KA, Chen X, Tzipori SR, Kumamoto CA (1999). Invasive lesions containing filamentous forms produced by a *Candida albicans* mutant that is defective in filamentous growth in culture. Infect Immun.

