# Supplementary material for: Functioning of a tripartite lignocellulolytic microbial consortium cultivated under two shaking conditions: a metatranscriptomic study
Source: Biotechnol Biofuels Bioprod. 2023 Mar 29;16:54. doi: 10.1186/s13068-023-02289-0 (PMC10061750; doi:10.1186/s13068-023-02289-0)
Supplement: Supplementary file 2 — Additional file 2: Table S2. Distribution of adhesion related genes of Coniochaeta sp. 2T2.1. Figure S1. Physical appearance of (a and d) bacterial–fungal consortium (Coniochaeta sp. 2T2.1, Sphingobacterium paramultivorum w15 and Citrobacter freundii so4) and (b and c) monoculture of Coniochaeta sp. 2T2.1 at 60 rpm (a and b) and 180 (c and d) rpm using wheat straw as the sole of carbon and energy source. Figure S2. Principal components analysis (PCA) of the expression profile of (a) Citrobacter freundii so4, (b) Sphingobacterium paramultivorum w15 and (c) Coniochaeta sp. 2T2.1. Figure S3. Growth indicator genes dynamics of (a) Sphingobacterium paramultivorum w15 and (b) Citrobacter freundii so4; (c) respiration regulating genes of Citrobacter freundii so4; (d) hyphal and (e) adhesion genes of Coniochaeta sp. 2T2.1 at 180 rpm (black) and 60 rpm (grey). Figure S4. Expression dynamics of peptidoglycan related genes in (a) Citrobacter freundii so4 and (b) Sphingobacterium paramultivorum w15. Figure S5. Expression dynamics of proteoglycans-attacking related genes expressed at 180 rpm (black) and 60 rpm (grey) in Coniochaeta sp. 2T2.1. Figure S6. Reactive oxygen species (ROS) protection genes in Sphingobacterium paramultivorum w15. Figure S7. Expression dynamics of detoxification related genes in Citrobacter freundii so4. Figure S8. Expression dynamics of biofilm and stress related genes in Citrobacter freundii so4. Figure S9. Phylogenetic tree of (a) bystin and (b) glycoproteins of Coniochaeta sp. 2T2.1. [file 13068_2023_2289_MOESM2_ESM.docx]

**Functioning of a tripartite lignocellulolytic microbial consortium cultivated under two shaking conditions: A metatranscriptomic study**

Yanfang Wang^1^*, Diego Javier Jiménez^2^, Zhenhua Zhang^3^, Jan Dirk van Elsas^1^*

1 Cluster of Microbial Ecology, Groningen Institute for Evolutionary Life Sciences, University of Groningen, Groningen, the Netherlands

2 Microbiomes and Bioenergy Research Group, Department of Biological Sciences, Universidad de los Andes, Bogotá, Colombia.

3 Department of Genetics, University Medical Center Groningen, Groningen, the Netherlands

*** Correspondence:**Jan Dirk van Elsas
j.d.van.elsas@rug.nl

Yanfang Wang

wang-yanfang@qq.com

1. Additional Data

**Expression of growth- and adhesion-associated genes**

***Expression at 180 rpm –*** As outlined in the foregoing, *S. paramultivorum* w15 grew rapidly early on and reached stationary phase as from middle growth stage. Both *ftsA* and *ftsZ* genes showed intermediate expression levels, of 116-200 TPM, from early to late stages, with minor decreases over time. Moreover, gene *rpoD* was found to be highly expressed at early (1,984 TPM) and middle (1,379 TPM) stages, indicating a considerable number of cells exhibiting growth. The expression decreased significantly (Log2FoldChange=1.4±0.3, *padj*<0.001), to 608 TPM, at late stage (Fig. S2a).

The expression of the *C. freundii* so4-specific *ftsA* and *ftsZ* genes was relatively stable, at intermediate levels, i.e. 101 - 155 TPM, over the experimental time (Fig. S2b). Remarkably, the expression of *bolA* was at intermediate level (393 TPM) at early stage, decreasing significantly (Log2FoldChange=1.1±0.1, *padj*<0.001) to 230 TPM (middle stage) and staying at this level at late stage. Also, the *elaB* gene was expressed at high level (2,473 TPM) in early stage, remaining high, albeit significantly (Log2FoldChange=1.24±0.2, *padj*<0.001) reduced (1,237 TPM) at middle and late stages. This indicated that a major part of the so4 cell population was in need of oxidative stress protection (spin-off of rapid metabolism), but that this became somewhat less dominant in later stages. The aerobic respiration regulatory gene *arcA* was expressed at around 1,072 TPM at early stage, and decreased gradually to 624 TPM at middle and 730 TPM at late stage. Finally, the anaerobic regulatory *fnr* gene was expressed at intermediate (184-382 TPM) levels, decreasing over time.

As for strain 2T2.1, the two *efg1* genes – involved in hyphal formation - were expressed at low levels (~46 TPM) at early stage, increasing to intermediate level at middle stage (~110 TPM), and again decreasing at late stage (~70 TPM). The two bystin genes (98.16% similarity - located in the same contig; Table S3) showed low expression levels over time (10-20 TPM) (Fig. S2e). Six of the eight genes encoding adhesion glycoproteins showed relatively stable expression levels over time (two between 2-20 TPM, and four between 100-200 TPM; Fig. S2e), with the remaining two being barely expressed.

***Expression at 60 rpm –*** At 60 rpm, the strain w15 *ftsA* and *ftsZ* genes showed intermediate expression levels (~300 TPM) at early stage, decreasing significantly (Log2FoldChange=1.3±0.1, *padj*<0.001) to 136 TPM, at middle stage and remaining at that level till late stage. The *rpoD* gene expression showed an overall decreasing trend, from 1,312 *(*early stage), via 874 (middle stage), to 492 TPM at late stage (significant decrease; Log2FoldChange=1.1±0.3, *padj*<0.001). The expression of gene *dps* was at high level (2,798 TPM) at early stage, staying at high levels (3,000- 4,000 TPM) at middle and late stages.

Regarding strain so4, the *ftsA* and *ftsZ* genes were expressed at intermediate levels over all experimental time points (133-186 TPM), indicating active subpopulations of so4 were growing across all time points (Fig. S2b). Gene *elaB* was expressed at high level (2,072 TPM) at early stage, decreasing significantly (Log2FoldChange=2.3±0.2, *padj*<0.001) to 492 TPM at middle stage, and again significantly (Log2FoldChange=1.2±0.2, *padj*<0.001) to 237 TPM at late stage. The so4 *bolA* gene was initially expressed at intermediate level, gradually decreasing over time, from 494 (early) to 254 TPM (late). The aerobic respiration regulatory gene *arcA* was expressed at around 1,234 TPM at early stage, decreasing to 789 TPM at middle and 474 TPM at late stages. The anaerobic regulatory gene *fnr* was expressed at 389 TPM early on, and fluctuated across this value in middle and late stages (range 221-427 TPM).

The two *efg1* genes in strain 2T2.1 were expressed lowly (~67 TPM) at early stage, staying at this level at middle stage, then increasing significantly (Log2FoldChange=0.5±0.2, *padj*<0.001) at late stage (~110 TPM). This dynamics indicated that strain 2T2.1 invested progressively more energy in hyphal formation (Fig. S2d). The two bystin genes were expressed at low levels, with an increase from early (~11 TPM) via 23 TPM at middle stage to late stage (~45 TPM). Six of the eight adhesion glycoprotein genes were expressed at low (2-71 TPM) to intermediate levels (110~400 TPM), with increases over time; two genes were significantly upregulated (Log2FoldChange=2.1±0.3, *padj*<0.001) from early to middle stages (from 4 to 29 TPM), increasing further at late stage (71 TPM) (Fig. S2e).

***Comparison between 180 rpm and 60 rpm*** – The comparative gene expression analyses across shaking speeds revealed that most of the strain w15 indicator genes (i.e. *ftsA,* *ftsZ* and *rpoD*) were expressed at similar levels (Log2FoldChange<1 or *padj*>0.05) at similar time points across the two shaking speeds. However, at late stage, gene *dps* was significantly less expressed (Log2FoldChange=2.0±0.2, *padj*<0.001) at 60 rpm (3,493 TPM) than at 180 rpm (12,759 TPM), which is consistent with the premise that (starvation) stress in strain w15 was lower at 60 rpm than at 180 rpm.

Furthermore, the *ftsA,* *ftsZ* and *bolA* genes in strain so4 were also expressed at similar levels (Log2FoldChange<1 or *padj*>0.05) across the two shaking speeds. However, *elaB* gene expression, starting at similar levels, was significantly lower at 60 than at 180 rpm at middle (Log2FoldChange=1.7±0.2, *padj*<0.001) and late stages (Log2FoldChange=2.9±0.2, *padj*<0.001), indicating less stressful conditions at 60 rpm (Fig. S2c).

The comparative analyses further showed that *Coniochaeta* sp. 2T2.1 expressed genes for hyphal formation and adhesion preferentially at 60 rpm, as compared to 180 rpm. For instance, the expression of the *efg1* genes, albeit at low level, was higher (Log2FoldChange=0.5±0.2, *padj*<0.05) at 60 than at 180 rpm at early and late stages. Moreover, at late stage the two bystin genes were significantly higher expressed (Log2FoldChange=1.7±0.2, *padj*<0.001) at 60 (60 TPM) than at 180 rpm (15 TPM).

1. Additional Tables

Table S2 Distribution of adhesion related genes of *Coniochaeta* sp. 2T2.1

The expression of one of the genes marked yellow in each cluster was shown in Fig. S2e.

Genes not marked yellow are the two genes barely expressed.

1. Additional Figures


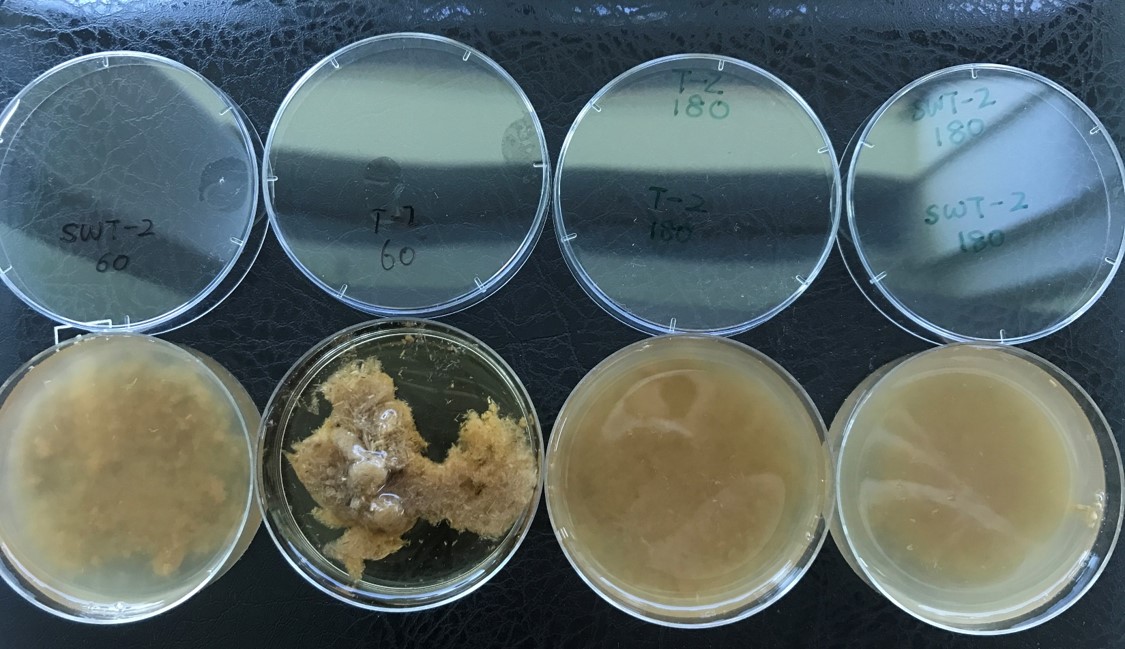


(a)

(d)

(c)

(b)


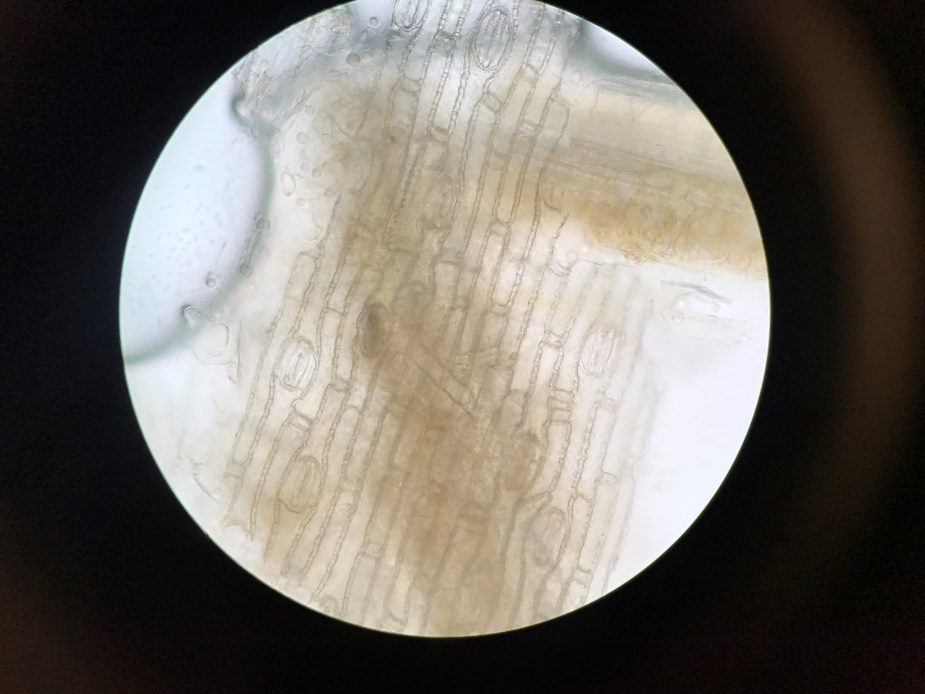

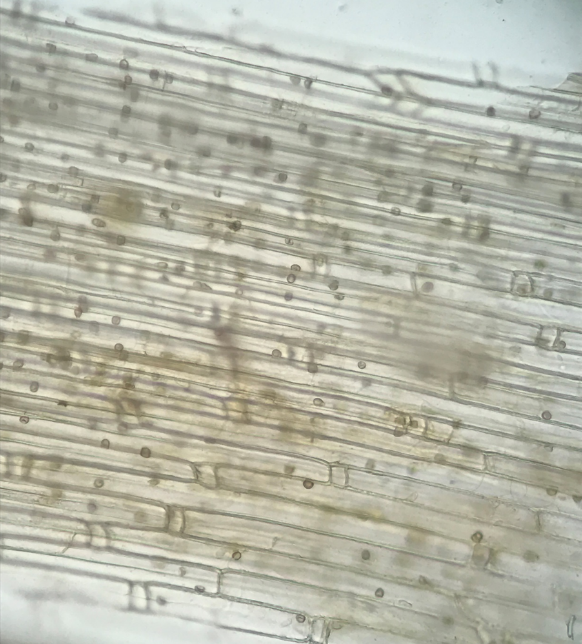


(f)

(e)

Fig. S1 Physical appearance of bacterial-fungal consortium (*Coniochaeta* sp. 2T2.1, *Sphingobacterium paramultivorum* w15 and *Citrobacter freundii* so4) and monoculture of *Coniochaeta* sp. 2T2.1 at 60 rpm (a consortium and b monoculture) and 180 rpm (c monoculture and d consortium); And bacterial-fungal consortium growing on wheat straw at (e) 60 rpm and (f) 180 rpm under 100X magnification.


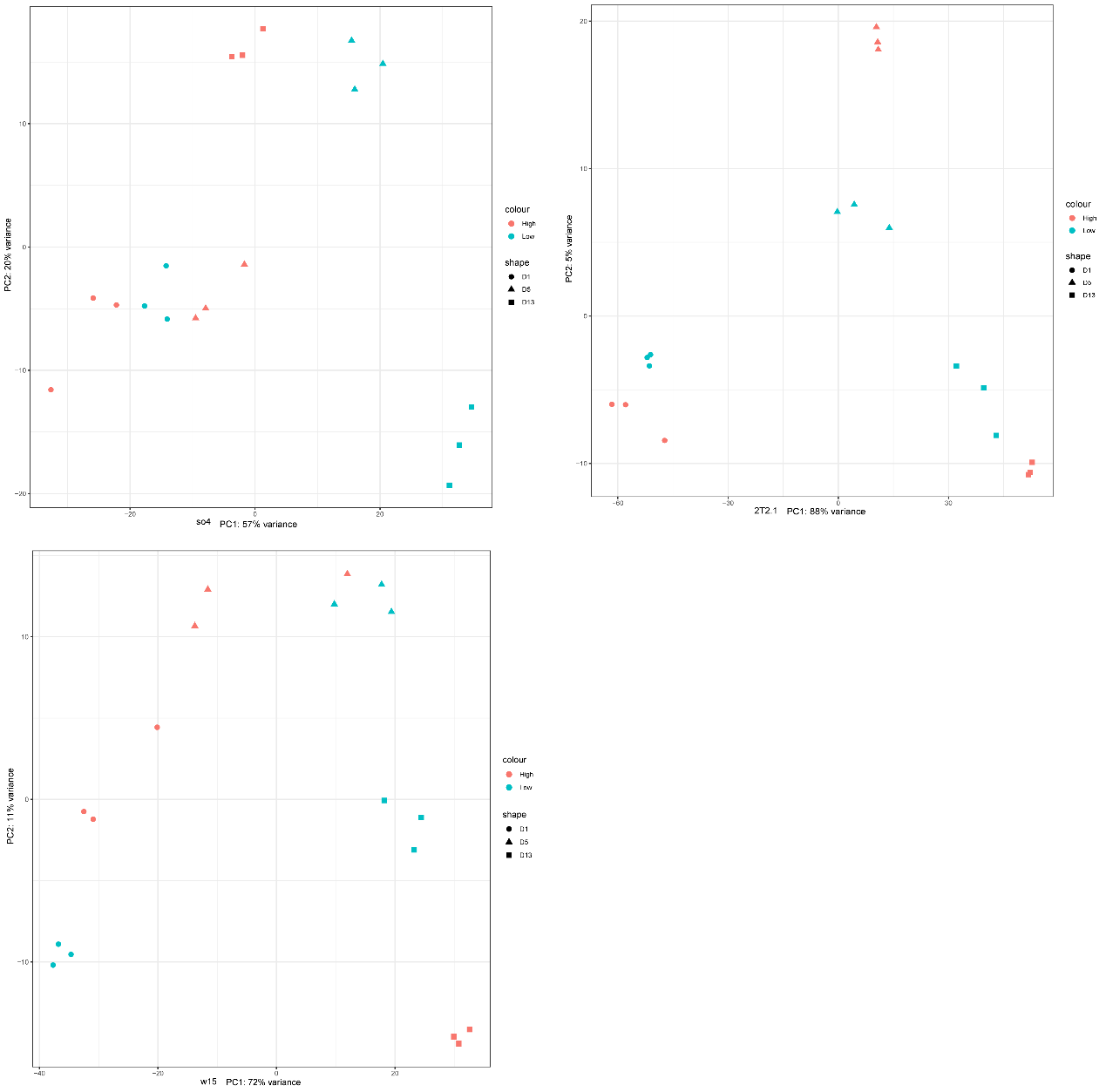


(c)

(b)

(a)

Fig.S2 Principal components analysis (PCA) of the expression profile of (a) *Citrobacter freundii* so4, (b) *Sphingobacterium paramultivorum* w15 and (c) *Coniochaeta* sp. 2T2.1; red: high shaking speed 180 rpm, blue: low shaking speed 60 rpm; circle: 1 day; triangle: 5 days; square: 15 days.


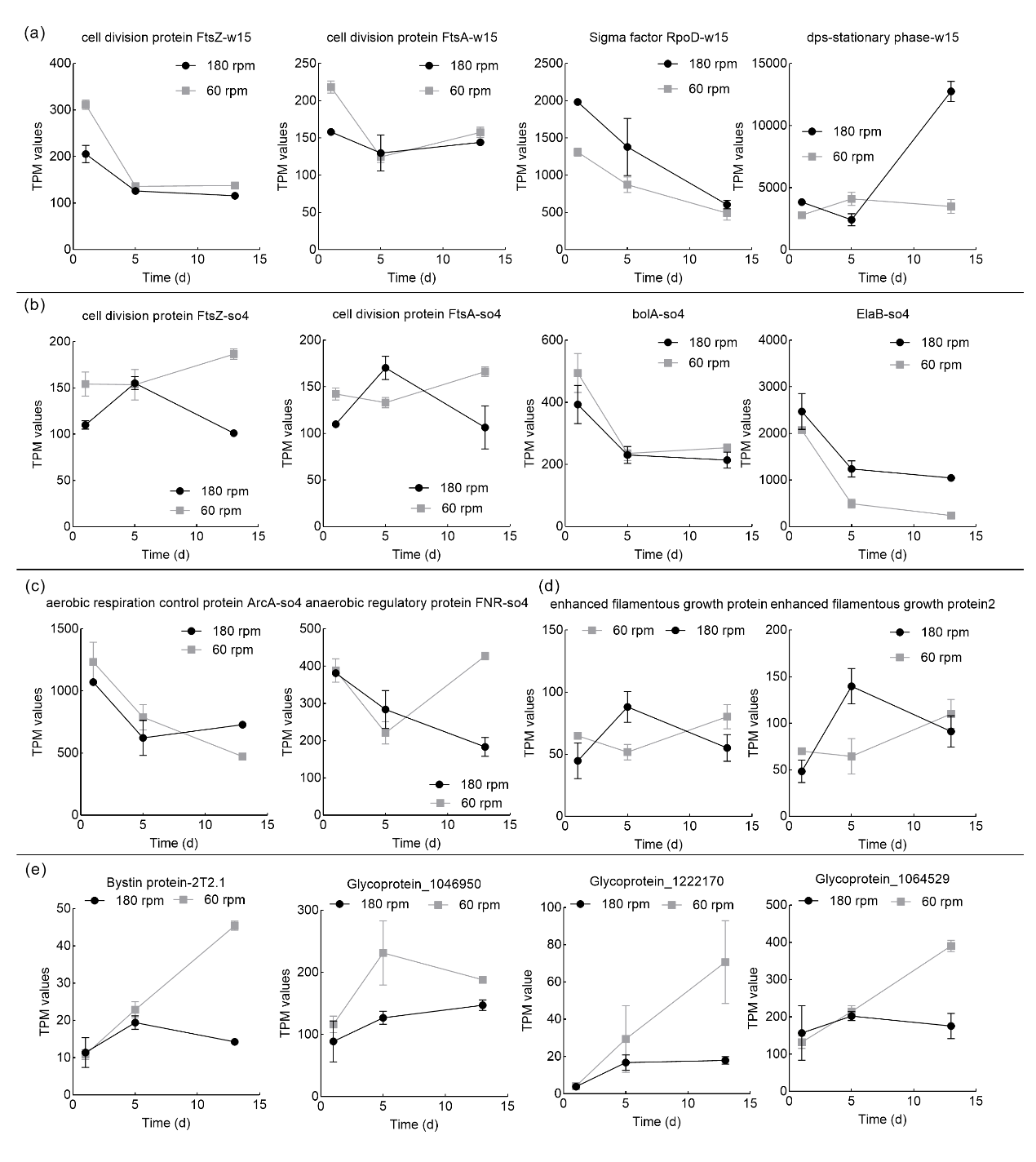


Fig. S3 Growth indicator genes dynamics of (a) *Sphingobacterium paramultivorum* w15 and (b) *Citrobacter freundii* so4; (c) respiration regulating genes of *Citrobacter freundii* so4; (d) hyphal and (e) adhesion genes of *Coniochaeta* sp. 2T2.1 at 180 rpm (black) and 60 rpm (grey)


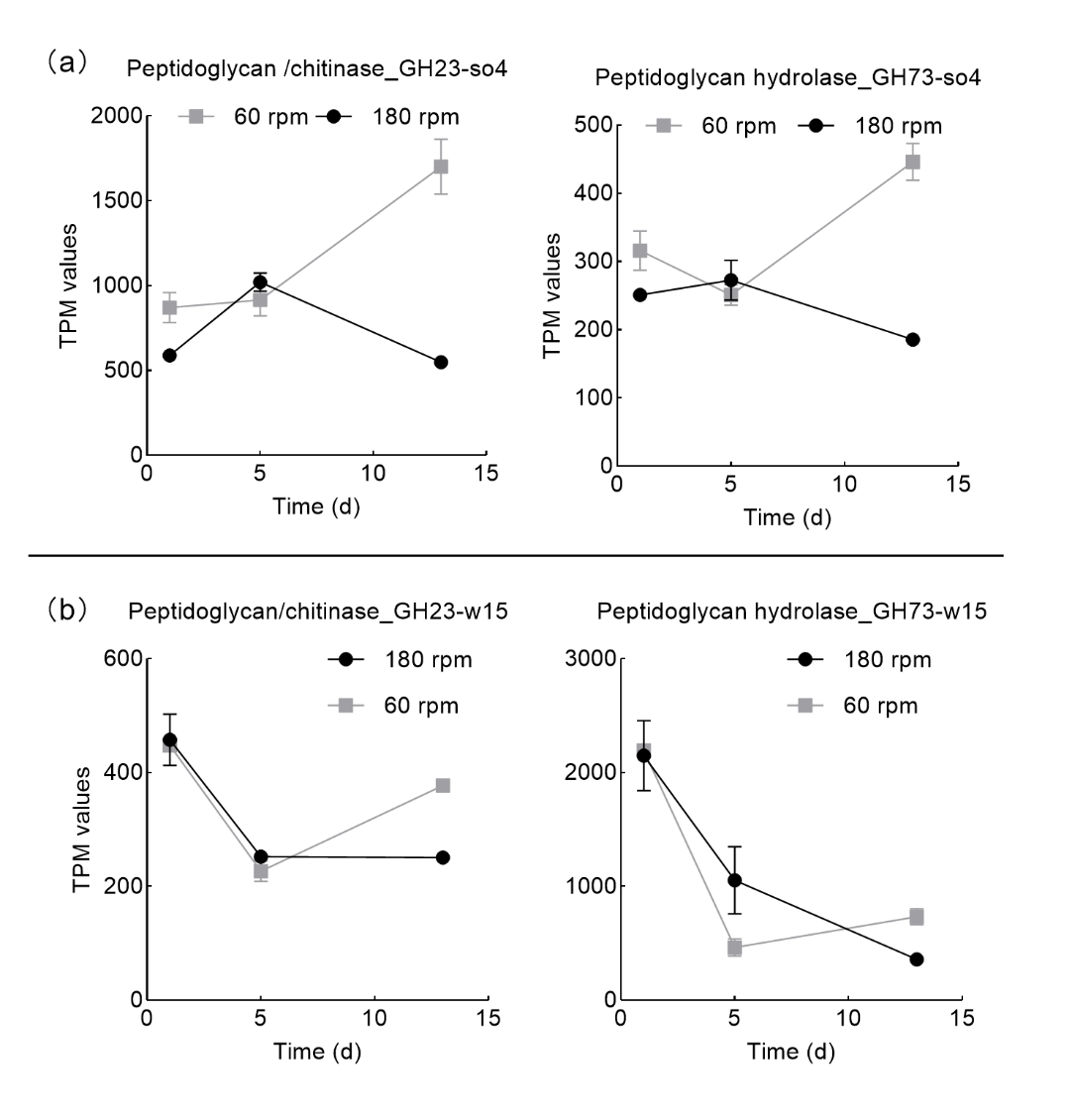


Fig. S4 Expression dynamics of peptidoglycan related genes in (a) *Citrobacter freundii* so4 and (b) *Sphingobacterium paramultivorum* w15


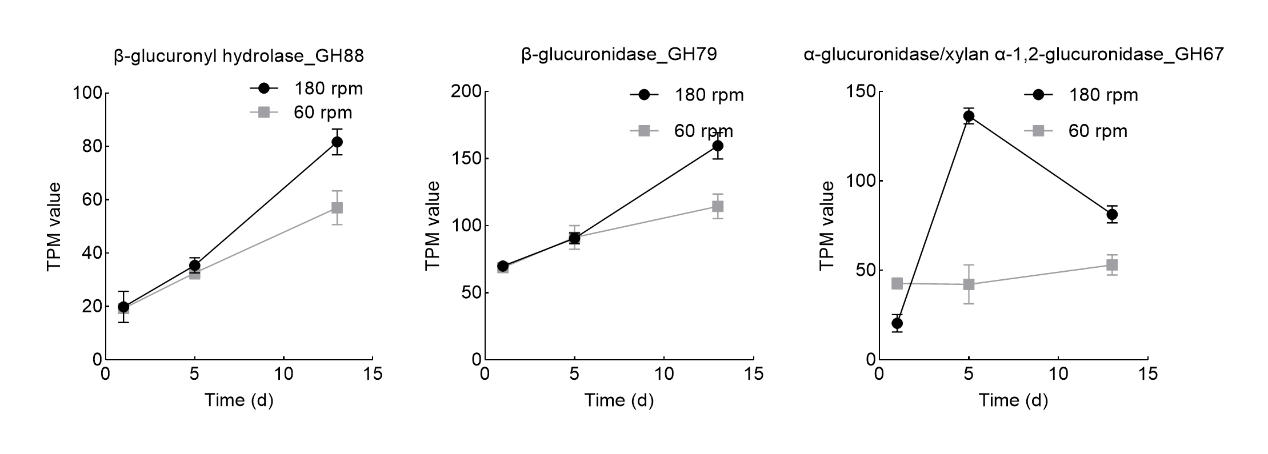


Fig. S5 Expression dynamics of proteoglycans-attacking related genes expressed at 180 rpm (black) and 60 rpm (grey) in *Coniochaeta* sp. 2T2.1


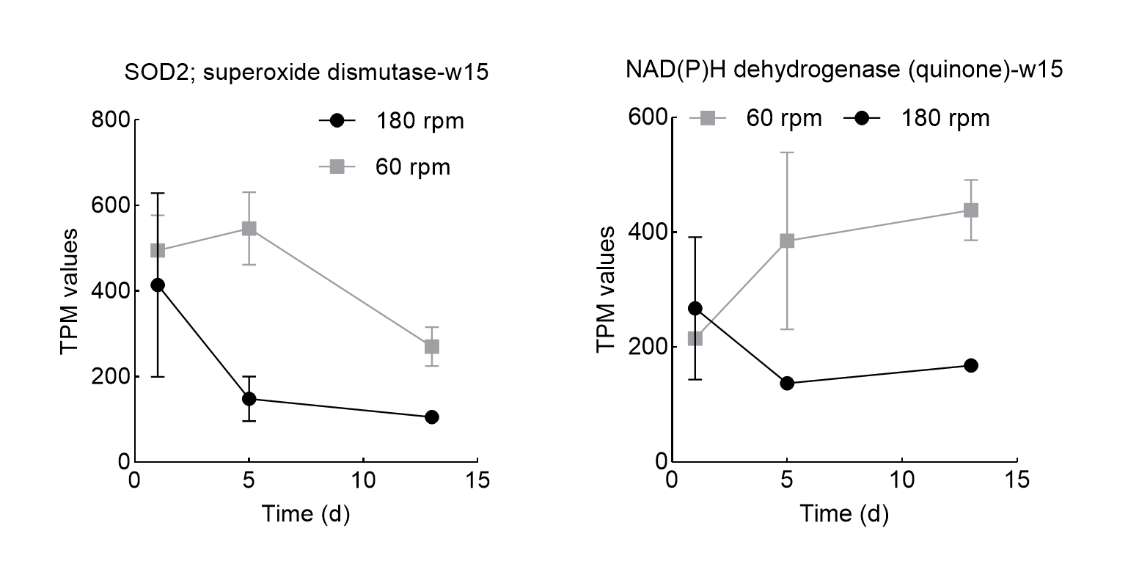


Fig. S6 Reactive oxygen species (ROS) protection genes in *Sphingobacterium paramultivorum* w15


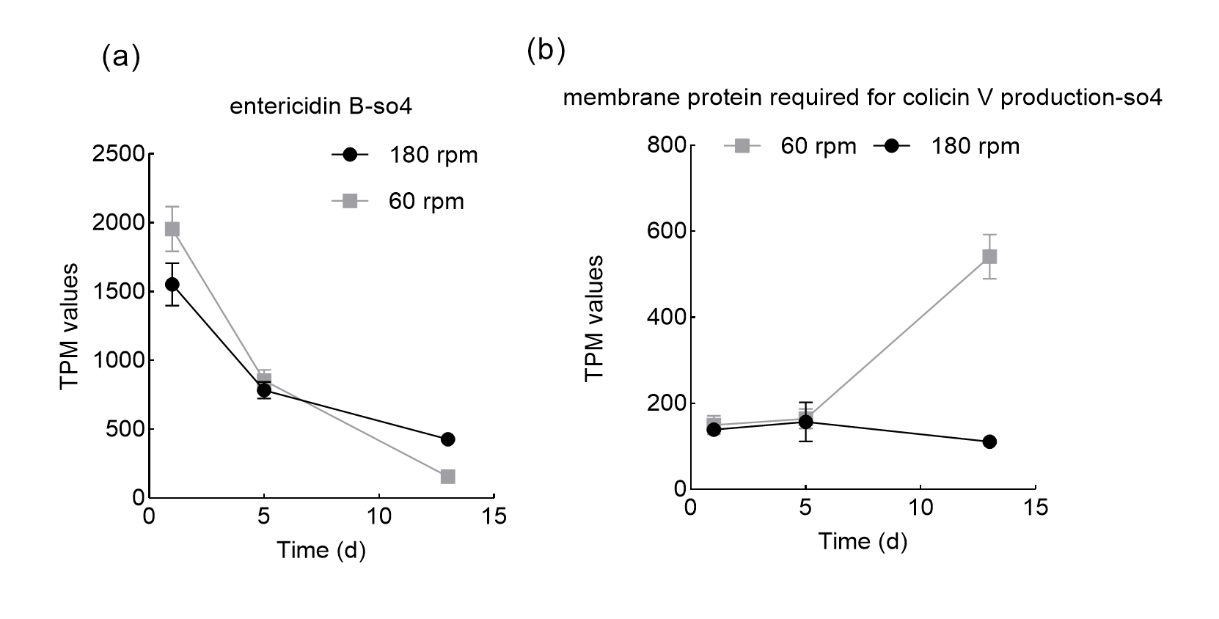


Fig. S7 Expression dynamics of detoxification related genes in *Citrobacter freundii* so4


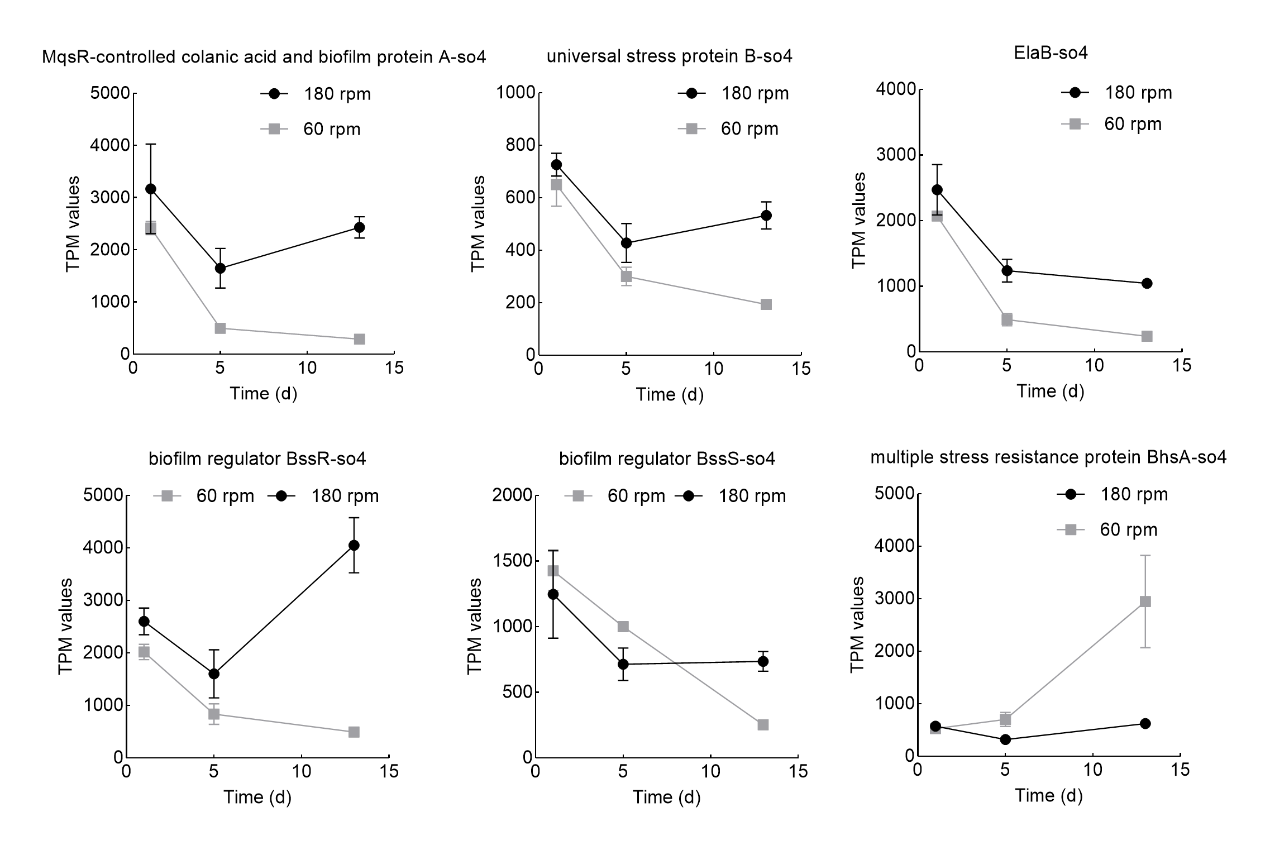


Fig. S8 Expression dynamics of biofilm and stress related genes in *Citrobacter freundii* so4


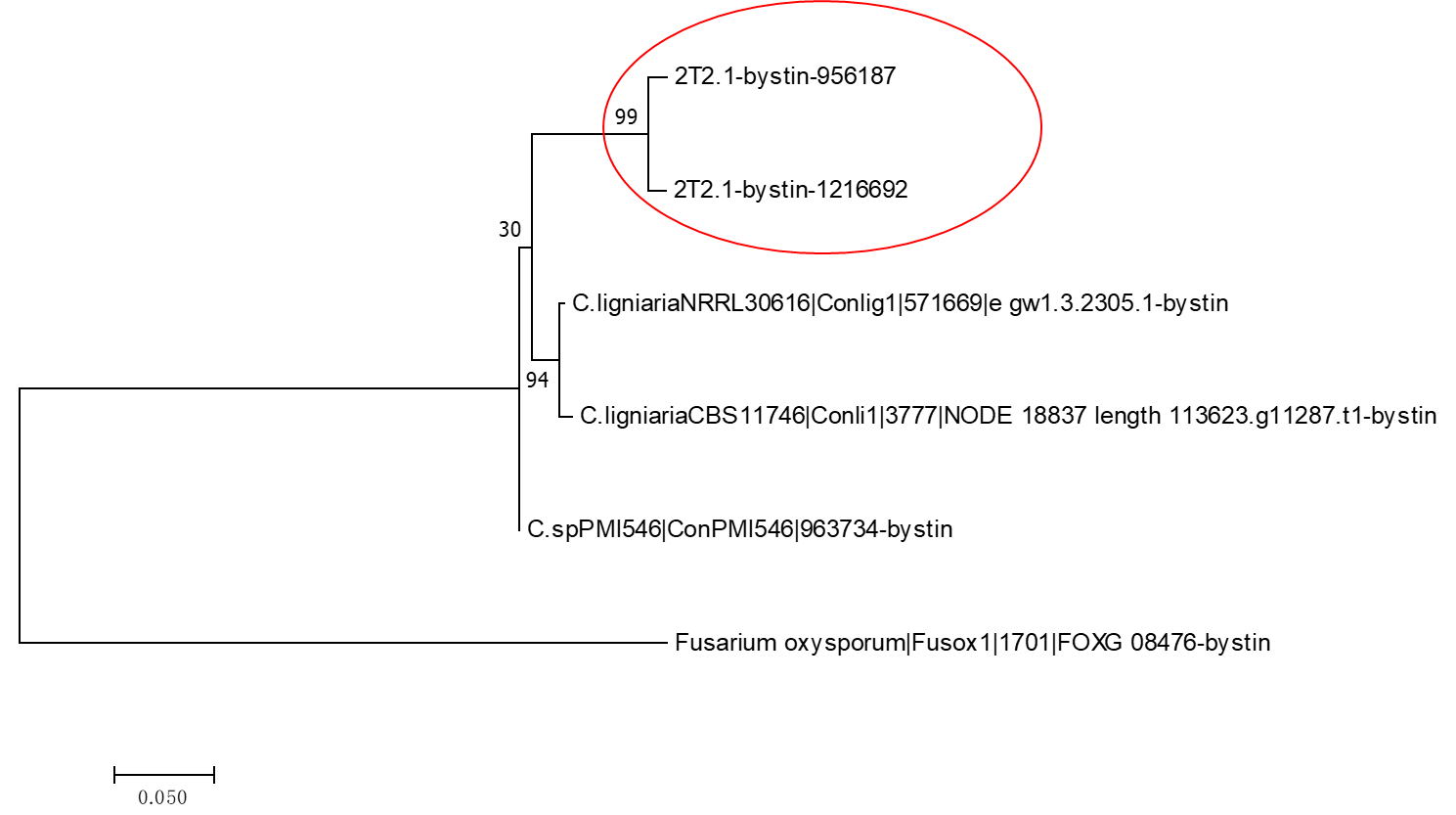


(a)


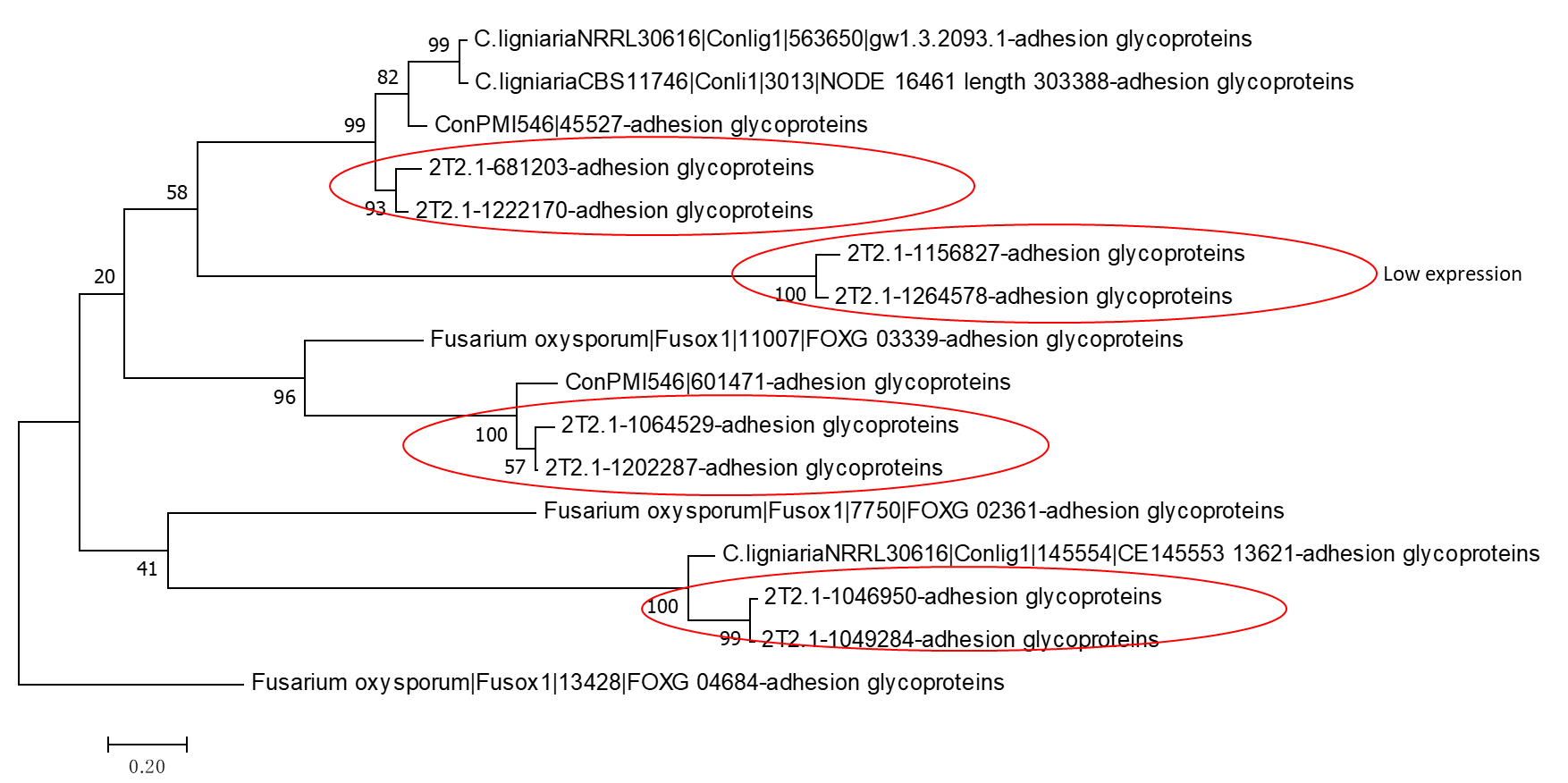


(b)

Fig. S9 Phylogenetic tree of (a) bystin and (b) glycoproteins of *Coniochaeta* sp. 2T2.1
